# Unexpected solvent effects on the UV/Vis absorption spectra of *o*-cresol in toluene and benzene: in contrast with non-aromatic solvents

**DOI:** 10.1098/rsos.171928

**Published:** 2018-03-14

**Authors:** Dong Zheng, Xiang-Ai Yuan, Haibo Ma, Xiaoxiong Li, Xizhang Wang, Ziteng Liu, Jing Ma

**Affiliations:** Key Laboratory of Mesoscopic Chemistry of MOE School of Chemistry and Chemical Engineering, Nanjing University, Nanjing 210093, People's Republic of China

**Keywords:** UV/Vis spectrum, aromatic solvent, solvent effect, *o*-cresol

## Abstract

Cresol is a prototype molecule in understanding intermolecular interactions in material and biological systems, because it offers different binding sites with various solvents and protonation states under different pH values. It is found that the UV/Vis absorption spectra of *o*-cresol in aromatic solvents (benzene, toluene) are characterized by a sharp peak, unlike the broad double-peaks in 11 non-aromatic solvents. Both molecular dynamics simulations and electronic structure calculations revealed the formation of intermolecular π-complexation between *o*-cresol and aromatic solvents. The thermal movements of solvent and solute molecules render the conformations of *o*-cresol changing between *trans* and *cis* isomers. The π-interaction makes the *cis* configuration a dominant isomer, hence leading to the single keen-edged UV/Vis absorption peak at approximately 283 nm. The free conformation changes between *trans* and *cis* in aqueous solution rationalize the broader absorption peaks in the range of 260–280 nm. The pH dependence of the UV/Vis absorption spectra in aqueous solutions is also rationalized by different protonation states of *o*-cresol. The explicit solvent model with long-ranged interactions is vital to describe the effects of π-complexation and electrostatic interaction on the UV/Vis absorption spectra of *o*-cresol in toluene and alkaline aqueous (pH > 10.3) solutions, respectively.

## 1. Introduction

The solvent effect plays a dominant role in many forefront areas such as synthetic chemistry, molecular biology and material design [1–3], because most of the chemical reactions as well as structure characterizations are performed in various solutions. The influence of the solvent on spectroscopic properties and reactivity was conventionally rationalized by molecular properties of the solvent, such as dipole moment, electronic polarizability, hydrogen bond (HB) donor and acceptor capability, intermolecular electrostatic interactions, etc. [4]. Recently, some novel types of intermolecular or intramolecular π-complexation interactions with participation of aromatic rings have attracted much attention in studying supramolecular structure and important biorecognition processes [4–10]. Special attention is given to two kinds of non-covalent interactions (NCIs) with aromatic rings, i.e. π-stacking and X-H/π(X = C, O, N, S) interactions, which are ubiquitous in biomolecules, such as aromatic amino acids and DNA bases. It was also claimed that, for large biomolecules, the fragments surrounding π-complexations may play the role of a solvent [11]. The π-stacking interaction is pivotal in determining performance efficiencies of optoelectronic devices such as organic light-emitting diodes and organic thin-film transistors [12–16]. Very recently, aromatic stacking was experimentally demonstrated to play the key role in controlling solvent-dependent nucleation in crystal growth [17]. The search for a database of X-ray crystal structures revealed a high occurrence of parallel displaced (PD) and T-shaped (T) stacking styles of aromatic rings [9,18–26], rationalized by electronic structure calculations [21,22,27–39].

The complicated solute–solvent and solvent–solvent interactions raised the challenge of direct detection of the existence of π-complexation between aromatic rings in solutions. Various infrared (IR) techniques have been adopted to characterize the formation or dissociation process of various solute–solvent complexes of phenol, benzene and their derivatives [21,40–46]. For example, the dissociation time for the phenol–benzene complex was approximately 8 ps in the mixed benzene–CCl_4_ solvents, which was tripled (24 ps) by adding two electron-donating methyl groups to the benzene [40]. It was conceived that the formation of the non-covalent π-structure is favoured by the methyl group [33]. Most of the experimental processes were based on the IR spectral shift of the XH (X = N, O, S) stretching mode of the complexes between an XH-substituted aromatic ring (X = N, O, S) and aromatic compounds [8,9,20,40,41,43,45,47], while the existence of transient π-complexation lacks clear fingerprints in vibrational spectra. In addition, the influence of π-complexation on the electronic absorption spectra of solutions has been rarely reported. To this end, *o*-cresol is selected in this work as a prototype solute to investigate the effects of non-covalent intermolecular interactions on the UV/Vis absorption spectrum, because such a molecule offers three typical units: a benzene ring (which is requisite for π-complexation), a pH-sensitive hydroxyl group (acting as either HB donor or acceptor in inter/intramolecular interactions) and a methyl group (which is a hydrophobic group and sensitive to π-interactions). Very recently, it was also demonstrated that the cresol unit could work as a biomarker for diagnostic purposes [48].

**Figure 1.**
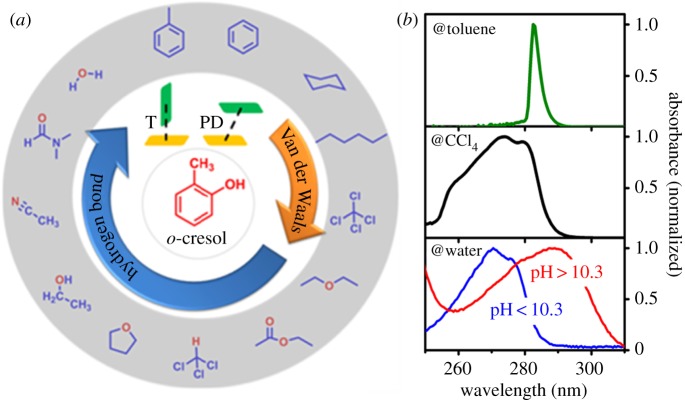
Schematic illustrations of (*a*) intermolecular interactions between solute *o*-cresol and 13 kinds of solvents, and (*b*) the difference in absorption spectra of solvated *o*-cresol in aromatic, non-polar and polar solvents, as well as the spectral difference under different pH conditions in aqueous solution. The stacking structures of T-shaped (T) and parallel displaced (PD) are also depicted in the inset.

Thirteen kinds of solvents, including both aromatic (toluene and benzene) solvents and non-aromatic ones (including water, *N*,*N*-dimethylformamide (DMF), acetonitrile, ethanol, tertrahydrofuran (THF), ethyl acetate, chloroform, diethyl ether, CCl_4_, hexane, cyclohexane) will be studied, as shown in figure 1. The interactions between the solute *o*-cresol and 13 solvents can be hence classified into three categories: π-complexation interaction in benzene and toluene solvent molecules; HB interactions in polar solvents, such as acetonitrile, THF and water; and van der Waals (vdW) interaction in non-polar solvents. It will be shown that owing to the existence of π-complexation, the single keen-edged peak of the absorption spectrum of *o*-cresol in an aromatic solvent is surprisingly different from those broad absorption spectra in other solutions with two humps. The combined molecular dynamics (MD) simulations and electronic structure calculations including density functional theory (DFT) and Møllet–Plesset theory (MP2) will provide a molecular-level insight into the intermolecular interactions between *o*-cresol and different solvents as well as the conformational change of *o*-cresol. The pH dependence of the absorption spectrum in aqueous solutions is also rationalized by our simulations on the neutral and protonated species. The present experimental and theoretical studies of the solvent effects in different solutions may shed light on regulation and control of molecular spectra of molecular aggregates in biomolecules and organic optoelectronic materials in various environments.

## 2. Details of computations and experiments

### 2.1. Measurements of UV/Vis absorption and infrared spectra

The *o*-cresol and all kinds of solvents used were purchased from commercial sources (analytical grade) and directly used in experiments without further purification. The UV/Vis spectra of *o*-cresol solutions were recorded with a Shimadzu UV-3600 double-beam spectrophotometer using a quartz glass cell with a path length of 10 mm (electronic supplementary material, figure S1) and 1 mm (electronic supplementary material, figure S2) at room temperature, respectively. To test the special interactions involved in aromatic solvents, the absorption spectra of the aromatic(toluene)–non-aromatic (CCl_4_) mixture solvent with a volume ratio of 1 : 1 were also detected (electronic supplementary material, figure S3).

The pH values of *o*-cresol aqueous solutions were adjusted in the range from 0.5 to 13.1 by the same procedures as used previously [49]. All pH measurements were carried out with a pB-10 digital pH meter (Sartorius, Germany) at room temperature. Several parallel and independent measurements of UV/Vis absorption spectra of *o*-cresol aqueous solution under different concentrations and pH conditions were also carried out.

Similar to what was done before [50,51], Fourier transform-IR (FT-IR) measurements were carried out on VERTEX70 FT-IR spectrometers (Bruker, Germany) for vapour samples (pure *o*-cresol and *o*-cresol/toluene mixture with a volume ratio of 1 : 1), as shown in the electronic supplementary material, figure S4.

### 2.2. Simulations of UV/Vis spectra in solutions

The polymer consistent force field (PCFF) [52–54] is employed in simulations of conformational changes using the explicit solvent model with the periodic boundary condition in different solutions of *o*-cresol. As for strong basic conditions (pH > 10.3) in aqueous solution, the neutral *o*-cresol is replaced by *o*-cresol anion and counterions Na^+^. The detailed information of the numbers of solute and solvent molecules, density and cell parameters are tabulated in the electronic supplementary material, table S1. The force field formulation, PCFF parameters for the water model and the validation of PCFF in describing the intermolecular interaction between *o*-cresol and water have been presented in our recent work [51].

MD simulations of *o*-cresol in various solutions were carried out in the canonical (NVT) ensemble at 298 K by using the Andersen thermostat [55]. The cut-off of vdW interactions is set to be 15.5 Å. The electrostatic interaction is evaluated by the Ewald summation. Motion equations were integrated by using the velocity Verlet algorithm with a time step of 1 fs. The 1 ns simulation in three typical kinds of solution (water, CCl_4_ and toluene) was subsequently carried out after the equilibrium state had been reached at 298 K. All MD simulations were performed with the discover module in the Materials Studio package [56]. From the MD trajectories, a series of snapshots of solute–solvent clusters were selected within 4 Å and 6 Å solvation shells to simulate UV/Vis spectra of *o*-cresol in toluene and aqueous solution, respectively.

The geometry optimization and frequency calculation of the gaseous structures, the vertical excitation energies of solute/solvent dimers and various clusters were carried out at various theoretical levels (electronic supplementary material, table S2) with the Gaussian 09 program [57]. The absorption spectrum of each conformation was displayed in either column form or the fitted curves by using a Gaussian function with a width of 0.2 eV at half height. The simulated UV/Vis absorption spectra were the statistical average of the calculated time-dependent DFT (TDDFT) spectra (with a scale factor of 1.1) of all the on-the-fly conformations sampled from MD trajectories.

The hybrid solvent model was employed in the TDDFT calculations. The long-ranged electrostatic interaction was described by using the polarizable continuum model (PCM) and the short-ranged solute–solvent interactions were considered in the explicit solvent models with the molecular clusters taken from the MD trajectories.

### 2.3. Analysis of non-covalent interaction

The intermolecular interaction energies, *E*_int_, between the solute (*o*-cresol) and solvent molecules were calculated by using equation (2.1):

2.1 $$E_{\mathrm{int}}=E_{\mathrm{solute}+\mathrm{solvent}}-E_{\mathrm{solute}}-E_{\mathrm{solvent}},$$

where *E*_solute+solvent_ and *E*_solute_ (*E*_solvent_) are the total energy of the molecular cluster of solute and solvent and the individual energy of an isolated solute (solvent) molecule, respectively. A more negative value of *E*_int_ represents a stronger intermolecular interaction between solute and solvent. In addition, natural bond orbital (NBO) [58] calculations were carried out to estimate the NBO charges and the orbital interaction energies between the bonding (donor) and anti-bonding (acceptor) orbitals. To illustrate the nature of NCIs such as steric repulsion, vdW interaction and the hydrogen bonding, the NCI [59] approach depending on the electron density and its derivatives was employed. NCIs were depicted by different colours using the Multiwfn [60] program for different solute and solvent clusters. From these grid points, the plotting colour-filled isosurfaces were obtained from the VMD program [61]. Zeroth-order symmetry-adapted perturbation theory (SAPT) [62] calculation was also performed on the open-source Psi4 electronic structure package to quantify the contributions of electrostatic and dispersion interactions for different complexes [63,64].

## 3. Results and discussion

**Figure 2.**
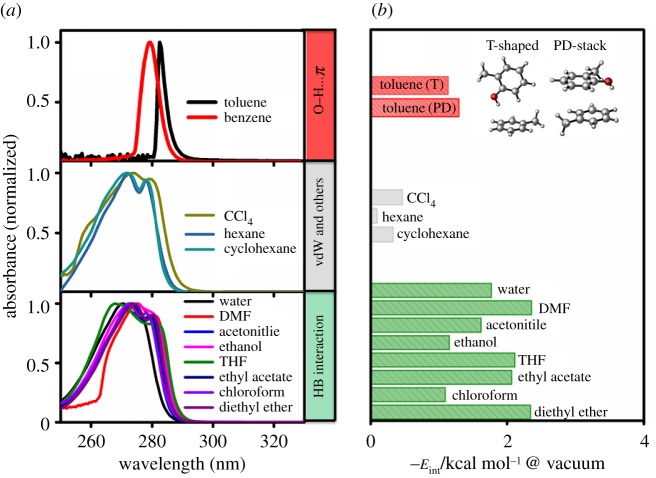
(*a*) The normalized UV/Vis absorption spectra of *o*-cresol in 13 kinds of solvents at the concentration of 3.5 × 10^−4^ M. (*b*) Intermolecular interaction energies, *E*_int_, for *o*-cresol with solvents in vacuum were calculated at the M06-2X/6-311++G(d,p) level with BSSE corrections. The structures of T-shaped and PD-stack *o*-cresol and toluene dimers are also depicted in the inset.

The solvation of *o*-cresol in aromatic (benzene and toluene) solvents leads to quite different optical properties: not only with the red shifts of the maximum absorption peak, but also with the extraordinary change in the shape of the absorption spectra (figure 2*a*). To the best of our knowledge, such a distinct difference in absorption spectra of *o*-cresol between aromatic and non-aromatic solvents has not been reported yet in the literature, although the interaction involving O–H…π (T-shaped: 1.15 kcal mol^−1^, PD-stack: 1.29 kcal mol^−1^) was weaker than HB interactions (1.10–2.37 kcal mol^−1^), as shown in figure 2*b*.The microscopic view underlying solvent effects on UV/Vis absorption spectra of *o*-cresol in different kinds of solvents will be presented through the in-depth study of the NCIs.

### 3.1. π-Complexation in aromatic solvents

**Figure 3.**
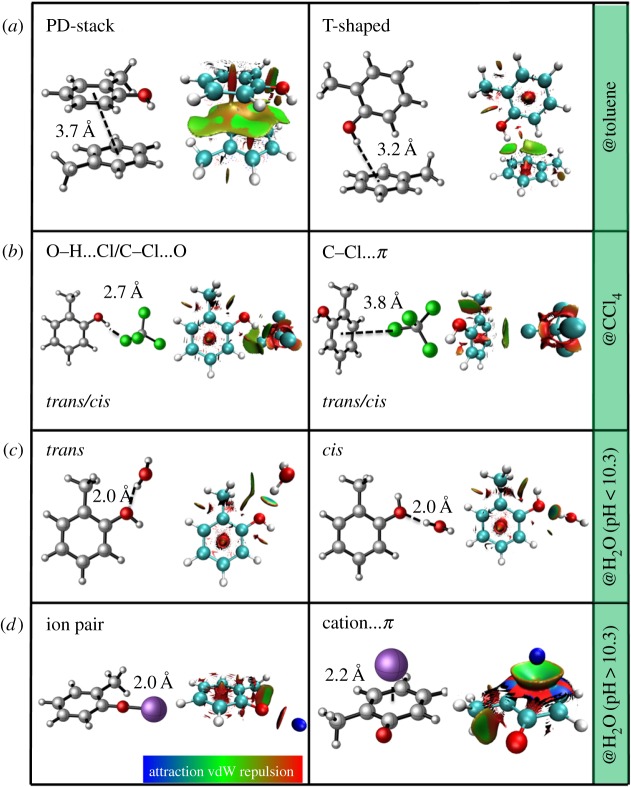
Different types of non-covalent interactions of (*a*) *o*-cresol@toluene, (*b*) *o*-cresol@CCl_4_, (*c*) *o*-cresol@H_2_O (pH < 10.3), and (*d*) *o*-cresol anion/Na^+^ (pH > 10.3). The geometries (except for the T-shaped dimer) were optimized at the M06-2X/6-311 + G(d,p) level. In the coloured isosurface plot, the green colour denotes a weak H-bond and the red colour stands for the steric effect.

To elucidate more clearly the NCIs between *o*-cresol and solvents, some typical dimers consisting of a *o*-cresol and solvent molecule (presented in figure 3) were optimized using both the M06-2X (table 1) and MP2 (electronic supplementary material, figure S6) methods with a 6-311 + G(d,p) basis set. It can be seen that the optimized geometries obtained from two different computational levels are very similar to each other, except that the T-shaped dimer can only be located at the MP2 level.

**Table 1.** The extent of charge transfer (CT) estimated from the difference in NBO charges of solute (*o*-cresol) and solvents, relative energy of the dimers, Δ*E*, intermolecular interaction energies, *E*_int_, orbital interaction energies, *E*_orb_, between the bonding and anti-bonding orbitals, and the maximum absorption peak, *λ*_max_, predicted by TDDFT (with a scale factor of 1.1).

	Δ*q*_CT_^a^ (*q*_solute_−*q*_solvent_)	*ΔE*^b^ (kcal mol^−1^)	*E*_int_^b^ (kcal mol^−1^)	*E*_orb_^a^ (kcal mol^−1^)	*λ*_max_^a^ (nm)
@toluene					
T-shaped	−0.006	0.0	−1.15	1.30 (π → σ*/*σ* → π*)	265
PD-stack	−0.008	1.4	−1.29	2.20 (π → π*)	270
@benzene					
T-shaped	−0.012	0.1	−1.17	3.14 (π → σ*/*n* → π*)	263
PD-stack	0.010	0.0	−1.18	0.90 (π → π*)	264
@CCl_4_					
C–Cl…π *trans*	0.004	0.0	−0.46	1.22 (π → σ*)	265
C–Cl…π *cis*	0.004	4.4	−0.47	0.99 (π → σ*)	265
O–H…Cl *trans*	−0.006	3.1	−0.40	1.18 (*n* → σ*)	264
C–Cl…O *cis*	0.004	5.1	−0.26	0.11 (*n* → σ*)	263
@H_2_O pH < 10.3					
*trans*	0.024	0.0	−1.13	3.64 (*n* → σ*)	261
*cis*	0.024	0.3	−1.11	4.90 (*n* → σ*)	261
@H_2_O pH > 10.3					
ion pair	—	0.0	—	—	310
cation…π	—	6.5	—	—	302

^a^The calculations were performed at the CAM-B3LYP/6-311 + G(d, p) level.

^b^The calculations were performed at the M06-2X/6-311 + G(d, p) level.

For *o*-cresol/toluene dimers (figure 3*a*), two energetically low-lying configurations are located at the MP2 level, namely, T-shaped (T) and PD, which are stabilized by the formation of an O–H…π interaction and π…π interaction, respectively. According to NBO analysis (table 1; electronic supplementary material, figure S5), the O–H…π interaction benefits from a favourable orbital interaction energy (denoted by *E*_orb_) between the bonding π (donor) of the aromatic solvent and the anti-bonding O–H orbital σ*_O–H_ (acceptor) of T-shaped *o*-cresol (*E*_orb_ = 1.3 kcal mol^−1^), slightly weaker than the π → π* interaction in the PD-stack (2.2 kcal mol^−1^). In a qualitative NCI map of *o*-cresol/toluene, the more attractive (green) part in the PD-stack dimer (figure 3*a*) is also more evident than that in the T-shaped dimer. SAPT energy components analysis of the interaction energy between *o*-cresol and toluene indicates that electrostatic attraction plays a dominant role in the intermolecular interaction in the T-shaped dimer (electronic supplementary material, table S3).

Upon π-complexation, an increase in electron density in these anti-bonding orbitals elongates the O–H-bond length (PD-stack: *r*_O–H_ = 0.969 Å; T-shaped: 0.966 Å) relative to that (0.963 Å) in the isolated *o*-cresol molecule. This is accompanied by small extents of charge transfer (CT) of 0.006 ∼ 0.012 e (table 1) between the solute and solvent fragments, close to that (less than 0.01 e) mentioned previously. [39] As shown in the electronic supplementary material, table S4, the aromatic molecules containing methyl groups are packed more closely (*o*-cresol/toluene centre-to-centre distance: 3.57 Å; phenol/toluene: 3.59 Å) than those without any methyl group (phenol/benzene: 3.63 Å; benzene/benzene: 3.66 Å). This trend is in good agreement with the relative order of interaction strength *E*_int_ (*o*-cresol/toluene:−1.29 kcal mol^−1^; *o*-cresol/benzene:−1.18 kcal mol^−1^; phenol/benzene: −1.03 kcal mol^−1^; benzene/benzene: −0.64 kcal mol^−1^). Similar to what was addressed previously [33], a conclusion is also presented here that the existence of the methyl group is favourable to form the π-complexation between aromatic systems. The bond orders from NBO analysis of *o*-cresol and solvents have been displayed in electronic supplementary material, figure S6. The change in bond orders is not evident because the intermolecular interactions between solute and solvents are rather weak.

As expected, the calculated *λ*_max_ of the PD-stack (270 nm) was slightly red-shifted than those of the T-shaped dimer (265 nm) and the isolated solute molecule (265 nm; electronic supplementary material, figure S10). The main absorption peak mainly comes from the π-π* transition of *o*-cresol (electronic supplementary material, figure S7). The adjacent toluene molecule is involved in the orbital transitions in some cases, especially for the PD-stack configuration. This implies that both short- and long-ranged interactions are crucial in reproducing the experimental spectra.

**Figure 4.**
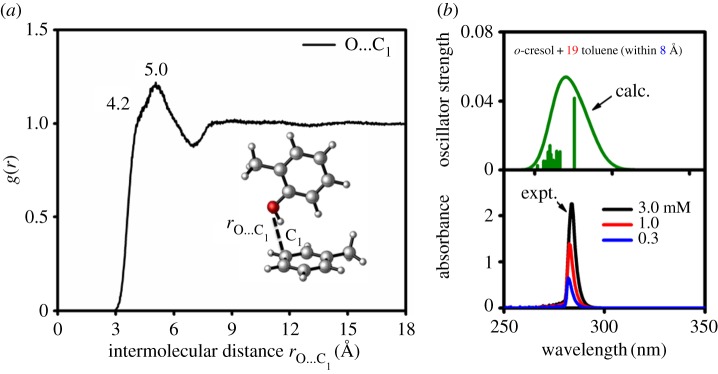
(*a*) The radial distribution function, *g*(*r*), for *r*_O…C1_ between the *o*-cresol oxygen atom (O) and the carbon (just under the OH group) in toluene. (b) The experimental (line) and simulated (column) UV/Vis absorption spectra of *o*-cresol with toluene solvents within 8 Å to consider the long-ranged interaction in toluene solutions. The absorption spectra were calculated at CAM-B3LYP/6-311 + G(d, p) level. The simulated absorption spectra are smoothed by a Gaussian function with a width of 0.2 eV at half height.

Shown in figure 4*a* is the radial distribution function (RDF) of distance *r*_O…C_ between the O and the C atom just below the OH group, which gives the onset position at 3 Å and the shoulder peak of 4.2 Å. The population of *r*_O…C_ within 3.0–4.2 Å indicates the formation of O–H…π interaction during the MD simulation of the solvent boxes, because the reported O–H…π interaction occurs at about 3.15 Å in ((4-bromophenyl)(diphenyl)methanol) crystal (CSD code: AHOSAT). The comparison between the RDF of *r*_O…π_ between *o*-cresol hydrogen atom (H) and the centroid of the aromatic ring of toluene and that of *r*_O…C_ in the CCl_4_ solvent is also given in the electronic supplementary material, figure S8. The nearest neighbour solute–solvent distances in aromatic solvents with the O–H…π interactions are smaller than those in the non-aromatic CCl_4_ solvent.

A series of molecular clusters (within 8 Å around the *o*-cresol hydrogen atom) were taken from the trajectory as exemplified by that at 500 ps (PD-stack) and 638 ps (T-shaped). The calculated absorption spectra of the clusters within 8 Å (290 nm) are red-shifted relative to the dimer clusters (270 nm) (electronic supplementary material, figure S9; table 1). Additional experiments were implemented to test the effect of π-complexation interaction on the UV/Vis absorption spectra in aromatic solvents. Shown in the electronic supplementary material, figure S2 are comparisons of the absorption spectra of *o*-cresol made between two series of experiments using different optical path lengths, 10 mm and 1 mm, respectively, from which the nearly identical optical properties are shown in toluene, water and CCl_4_ at the same concentration (5.6×10^−4^ M). The UV/Vis absorption spectrum of the toluene/CCl_4_ mixture solution is close to the one in toluene solvents except for little shifts of the maximum absorption position (electronic supplementary material, figure S3).

It should be mentioned, however, that there is an alternative interpretation of the spectra of *o*-cresol in aromatic solvents. The concentration dependence in absorbance (figure 4*b*) may also result from an additional absorbance originating from the dissolved *o*-cresol at a wavelength of approximately 280 nm. However, at lower wavelengths, the absorbance including solvent and solute reaches the maximum value at which detector saturation occurs much earlier with the additional contribution from the solute (*o*-cresol), which leads to the red shift of the maximum absorption peak. This interpretation is in line with the absorption spectra measured in cuvettes with a much shorter path length (1 mm). These spectra (electronic supplementary material, figure S2) show that the onset of the red edge of the absorption band (and not the whole band) can be measured down to slightly lower wavelengths, because the total absorbance (solvent plus solute) is much less in the shorter cuvette and thus does not ‘saturate the detector’. The sharp absorption bands are still unknown for organic molecules in condensed matter. More in-depth study of the solvent effect on absorption spectra is highly desired for future studies.

### 3.2. Dominant *cis* conformation in aromatic solutions versus free rotation of hydroxyl group in non-aromatic solutions

When *o*-cresol is solvated in non-polar solvents (CCl_4_), both intermolecular interaction energies (*E*_int_: -0.26 ∼ −0.46 kcal mol^−1^) and the extent of CT (0.004 ∼ 0.006 e) between solute and solvent were smaller than those in aromatic solvents. The weak non-bonded interaction is dominated by C–Cl…π interaction between the bonding orbital π (donor) of *o*-cresol and anti-bonding C–Cl orbital σ*_C–Cl_ (acceptor) as well as O–H…Cl interaction between the lone pair orbital of O (*n*_o_) and anti-bonding C–Cl orbital σ*_C−Cl_ (table 1; electronic supplementary material, figure S5). The intermolecular interaction in the solute–solvent pair is too weak to affect the excitation energy (*λ*_max _= 263 ∼ 265 nm) compared with isolated *o*-cresol (electronic supplementary material, figure S10). The maximum absorption peak (*λ*_max_) mainly comes from the π → π* transition of *o*-cresol, which is accompanied by CT between *o*-cresol and CCl_4_ (electronic supplementary material, figure S7). It is difficult to rationalize the difference of absorption spectra between aromatic and non-aromatic solvents just from the study of the dimer model. Hence, a statistic average of the *trans/cis* conformation of *o*-cresol will be analysed in the following based on the MD trajectories.

**Figure 5.**
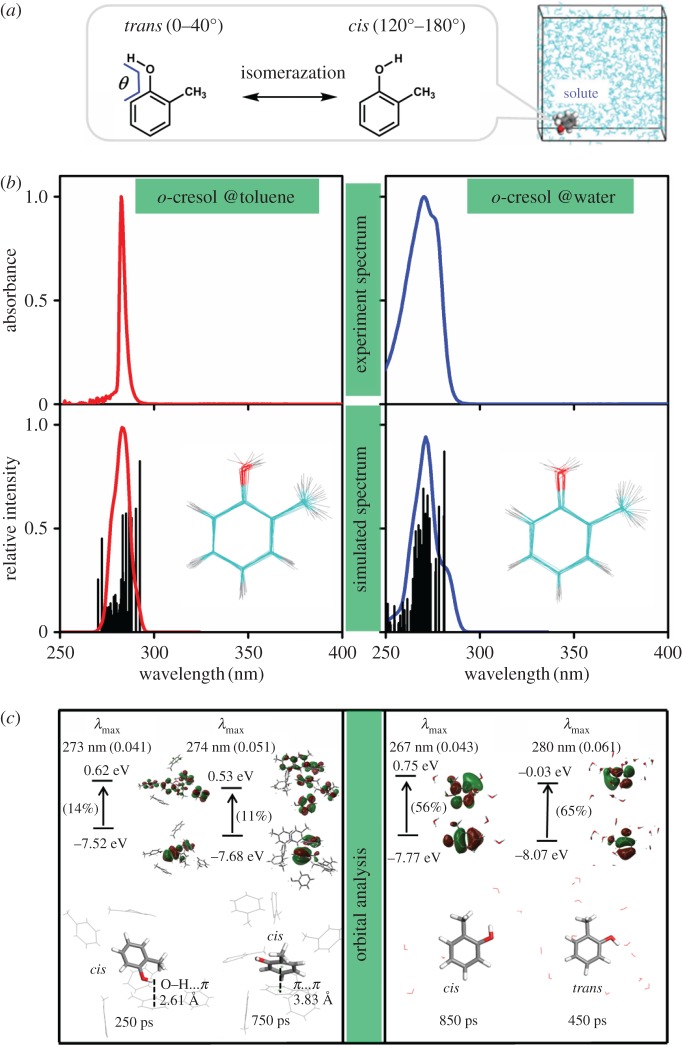
(*a*) The definition of the isomers of *o*-cresol based on the torsion angle *θ* in solutions. (*b*) The experimental and simulated spectra (with a scale factor of 1.1) of *o*-cresol in toluene and aqueous solution. The view of the aligned *o*-cresol molecule is presented in the inset. (*c*) The orbital analysis of a typical snapshot in toluene (250 ps and 750 ps) and in aqueous (450 ps and 850 ps) solution.

In neutral *o*-cresol, the torsion angle, *θ*_C–C–O–H_, termed *θ* in figure 5, was used to describe various rotational isomers of *o*-cresol. The emphasis is laid on two typical conformations of *o*-cresol, defined by the orientation of the proton in the phenolic hydroxyl group, *trans* (*θ* = 0 ∼ 40°) and *cis* (120° ∼ 180°), respectively. The electronic energy of the *trans o*-cresol isomer is slightly lower than that of the *cis* one in vacuum [51,61] CCl_4_ and H_2_O (table 1). Owing to the thermal motions of solvent and solute molecules in solutions, it is easy to overcome the rotational barrier between *trans* and *cis* isomers in *o*-cresol, and subject to the dynamic conformation changes upon solvation in various solvents [51]. In some other systems [65], the conformation population can be changed by NCIs and the energetically high-lying isomer of the solute may exist in a condensed phase.

In the vapour IR experiment spectra of pure *o*-cresol and solvated *o*-cresol in toluene (presented in the electronic supplementary material, figure S4), the absorption bands at about 2936 cm^−1^ were attributed to the stretching mode of the methyl group, and in this area such a stretching mode was missing in the spectrum of pure *o*-cresol. The OH stretching mode in the *o*-cresol dimer (3565 cm^−1^ and 3708 cm^−1^) and the *o*-cresol/toluene dimer (3654 cm^−1^) are low-frequency shifted relative to that in the *o*-cresol monomer (3720 cm^−1^).

The IR spectrum of *o*-cresol in toluene is quite close to that of aqueous solutions. However, the UV/Vis spectrum of the toluene solution is surprisingly different from that of the aqueous solution. To understand such a different solvent effect from an aromatic solution, we carried out a series of TDDFT calculations on the sequential 33 snapshots (being sampled from 200 ps to 1 ns MD simulations with a time interval of Δ*t* = 25 ps) in toluene and aqueous solutions, respectively. The details are presented in references [66,67] and the electronic supplementary material. The spectral line widths at half height are also progressively adjusted from 600 cm^−1^ (0.074 eV), 800 cm^−1^ (0.099 eV) to 1000 cm^−1^ (0.124 eV), as shown in the electronic supplementary material, figure S11.

In figure 5, a statistic average of all the calculated spectra of the snapshots reproduced the shape of the experimental spectrum in both toluene and aqueous solutions. The view of the superposition of the selected solute conformations in MD simulations revealed different percentages of *trans* or *cis* isomers of *o*-cresol in toluene and aqueous solutions (inset in figure 5*b*). The results reveal that the percentage of the *cis* conformation (78%) in toluene is higher than that (60%) in aqueous solution (electronic supplementary material, figure S12). The dominant *cis* conformation may be frozen by the π-interaction in aromatic (toluene) solution, hence the statistic average of the TDDFT spectra in toluene solution was a sharp keen-edged curve. The typical O–H…π and π…π interaction is exemplified by the snapshots at 250 ps and 750 ps in toluene solution, respectively. The orbital analysis also reveals that π interaction is involved in the transitions from the solute to the surrounding solvent molecules. As described in the electronic supplementary material, figure S13, the order parameters could describe the average orientation of solvent in solutions. In the bulk solution, the solvent was in isotropic orientation around the solute. The value of the order parameter increased significantly in the first solvation shell, it was conceived that local π-interaction could regulate the orientation of solvents.

In aqueous solutions, the calculated spectra of water surrounding *cis* (850 ps: *λ*_max_ = 267 nm,) and *trans* (450 ps: *λ*_max_ = 280 nm) isomers of *o*-cresol differ significantly in the maximum absorption peak. The different populations of *cis* (60%) and *trans* (20%) may cause the two overlapped peaks at 267 nm and 280 nm, respectively, with the latter one weaker in strength. Such an explanation also works for the *o*-cresol/CCl_4_ system.

### 3.3. pH dependence of UV/Vis spectra in aqueous solution

**Figure 6.**
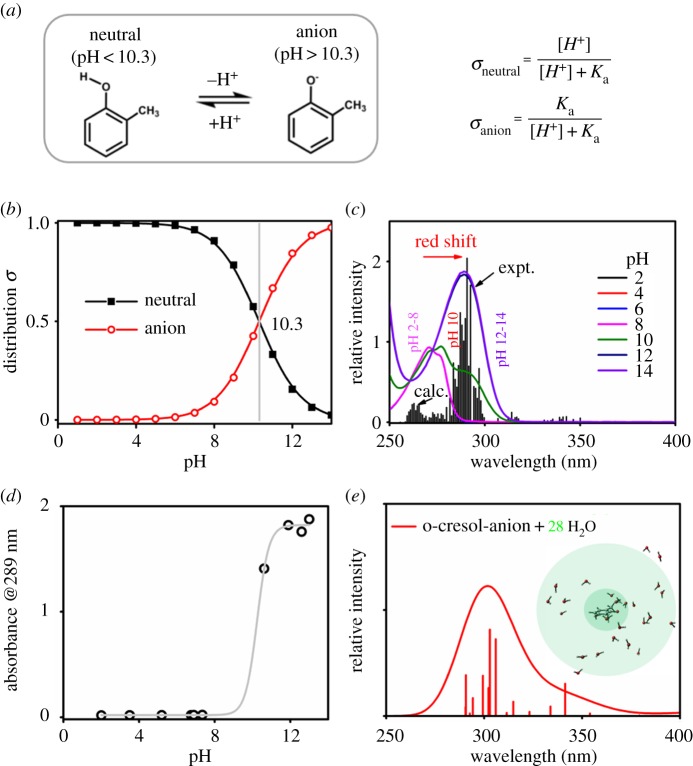
(*a*) The acid–basic equilibrium of *o*-cresol and the formulae of *δ*, *Ka* and pH values. (*b*) Distribution coefficient *δ* of *o*-cresol, *o*-cresol anion at different pH values. (c) The experimental UV/Vis absorption spectra (line) of 0.6 mM *o*-cresol in aqueous solutions at various pH values and the simulated UV/Vis spectra (column) based on the distribution coefficient, *δ*, of neutral *o*-cresol and *o*-cresol anion at different pH values. (*d*) Absorbance at 289 nm versus pH values. (*e*) The calculated UV/Vis absorption spectra (column) of *o*-cresol anion at 552 ps with solvent water within 8 Å (28 H_2_O) around the oxygen atom of the *o*-cresol anion.

With the presence of a hydroxyl group, the UV/Vis absorption spectra of *o*-cresol were sensitive to not only the solvents (figure 2) but also the pH values (figure 1*b*). The *pKa* of *o*-cresol in acidic conditions was reported to be 10.3 at 25.0°C [68]. The distribution coefficients of neutral (*δ*_neutral_) and anion (*δ*_anion_) forms of *o*-cresol can be calculated with *K*a and pH values, as shown in figure 6*b*. In a high-pH condition, *o*-cresol is deprotonated. The loss of H of the OH group makes the protonated *o*-cresol have only one possible conformation (in contrast with the *trans* or *cis* conformation of neutral *o*-cresol when pH < 10.3). The distance between the O anion and the hydrogen atom in the methyl group of *o*-cresol is 2.8 Å, suggesting little possibility of forming intramolecular HB interactions. The strong intermolecular interactions have been shown in the electronic supplementary material, figure S14, which shows the formation of intermolecular HB interactions (with *r*_O…H_ ≈ 2.03 and ∠O…H–O ≈ 160–172°) under a high-pH condition.

The *λ*_max_ of neutral *o*-cresol appeared at approximately 270 nm; however, when pH > 10.3, the *λ*_max_ of *o*-the cresol anion was red-shifted to 289 nm. According to the distribution coefficients of neutral and anion forms of *o*-cresol, we simulated the absorption spectra under different pH values (figure 6*c*). A closer look at the absorption intensity at 289 nm is then given in figure 6*d*, from which a steep climb of absorbance @289 is found when the pH value is larger than 10. Such a trend fits well with the increasing population of *o*-cresol anions as the pH value rises (pH = 10: *δ*_neutral_ = 0.67, *δ*_anion_ = 0.34; pH = 12, *δ*_neutral_ = 0.01, *δ*_anion_ = 0.99).

The long-ranged interaction [69] is also very significant in the aqueous solution under basic conditions. A good agreement between the calculation and experiment can be achieved by selecting a large solvation cluster involving 28 water molecules which are within 8 Å around the oxygen atom of the *o*-cresol anion, as shown in figure 6*e* and the electronic supplementary material, figure S14. As the adjacent water molecule is involved in the orbital transitions (electronic supplementary material, figure S15), the consideration of long-ranged electrostatic interaction yields a red shift of approximately 30 nm in *λ*_max_. The evolution of the Na^+^…O^−^ distance in 10 ns MD simulation (electronic supplementary material, figure S16) revealed that it is difficult to form ion pairs or cation/π-interactions in dilute solution under basic conditions.

## 4. Conclusion

For the first time, to our knowledge, the difference (change of shape and red shift of the *λ*_max_) in UV/Vis spectra of *o*-cresol between aromatic and non-aromatic solvents is experimentally observed. The combined MD simulations and electronic structure calculations demonstrated that the π-complexation interaction between *o*-cresol and aromatic solvents and the dominant *cis* conformation are responsible for the unique sharp keen-edged shape of the UV/Vis absorption spectra. By contrast, the comparable percentages of *cis* and *trans* isomers give rise to the two overlapped peaks in non-aromatic solutions. The combination of the implicit PCM model with explicit medium-sized solvent molecules around the solute molecule is crucial to reproduce experimental UV/Vis spectra by taking both the short- and long-ranged interactions into consideration. The insights into the conformation changes of the prototype solute *o*-cresol and the NCIs between *o*-cresol and various solvent molecules are useful in further regulation of the molecular spectra of molecular aggregates with regard to applications in biomolecules and various environments.

## Supplementary Material

SI for Royal-Society-Open-Science-submitted-20171115

## Data accessibility

The datasets supporting this article have been uploaded as part of the electronic supplementary material.
