# Supplementary material for: Unexpected solvent effects on the UV/Vis absorption spectra of o-cresol in toluene and benzene: in contrast with non-aromatic solvents
Source: R Soc Open Sci. 2018 Mar 14;5(3):171928. doi: 10.1098/rsos.171928 (PMC5882718; doi:10.1098/rsos.171928)
Supplement: SI for Royal-Society-Open-Science-submitted-20171115 [file rsos171928supp1.doc]

**Supporting Information**

#### Unexpected Solvent Effects on the UV/Vis Absorption Spectra of *o*-Cresol in Toluene and Benzene: In Contrast to Various Nonaromatic Solvents

*Dong Zheng,* **^†^** *Xiang-Ai Yuan,* **^†^** *Haibo Ma, Xiaoxiong Li, Xizhang Wang, Ziteng Liu, Jing Ma**

Key Laboratory of Mesoscopic Chemistry of MOE School of Chemistry & Chemical Engineering, Nanjing University, Nanjing, 210093, People’s Republic of China

^†^D.Z. and X.Y contributed equally to this paper.

**Contents**

Contents

S1. UV/Vis Absorption Spectra.

S1.1 Experimental UV/Vis Spectra in Various Conditions. S3

S2. Experimental and Calculated IR Spectra.

S2.1 The Calculations for IR Spectra. S5

S3. Computational Details

S3.1 Detailed Information of MD Simulations. S6

S3.2 Details of Energy Decomposition. S8

S3.3 Simulated UV/Vis Spectra in Different Solutions.

S3.3.1 Cluster Model for Simulating UV/Vis Spectra in Solutions. S11

S3.2.2 The Combined MD and TDDFT Approach for Simulating UV/Vis Spectra in Solutions. S13

S4. Cartesian coordinates of of all involved molecular structures

**S1. UV/Vis Absorption Spectra.**

**S1.1 Experimental UV/Vis Spectra in Various Conditions.**


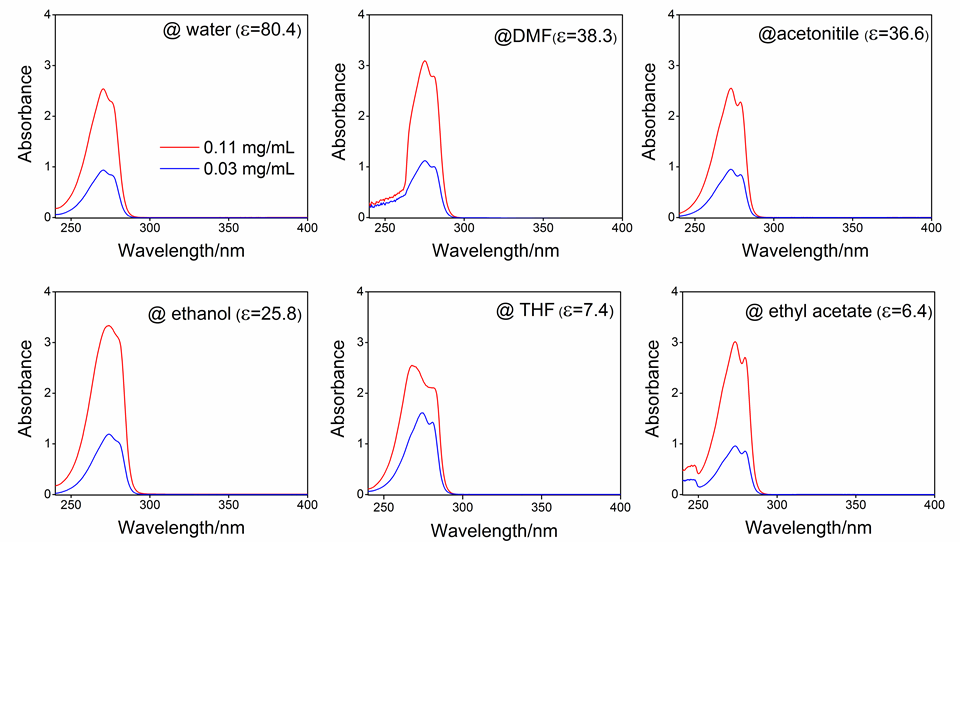


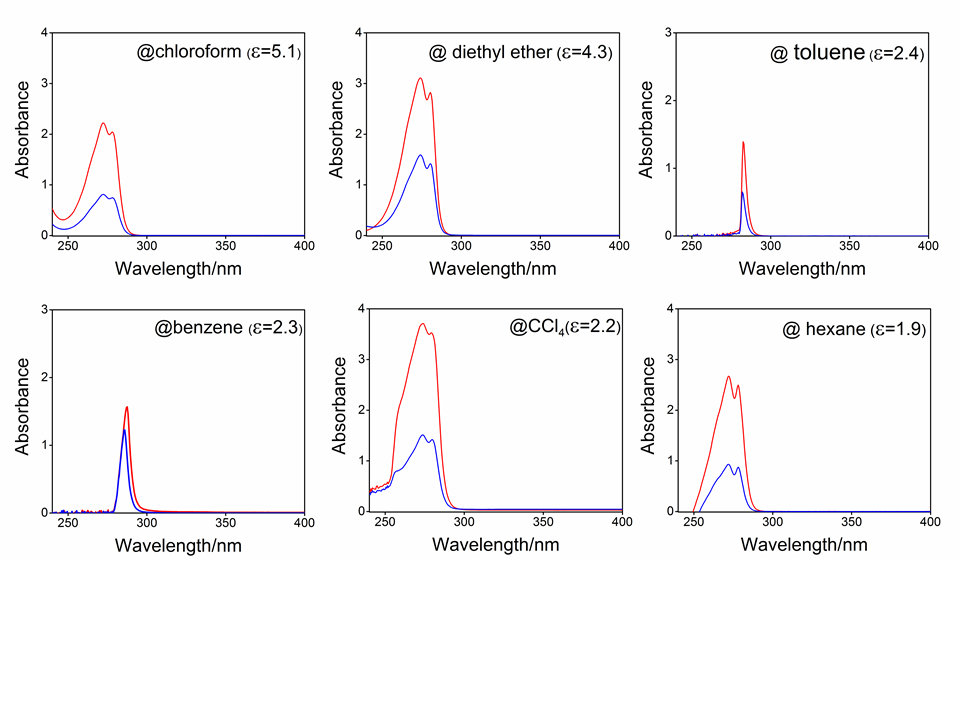


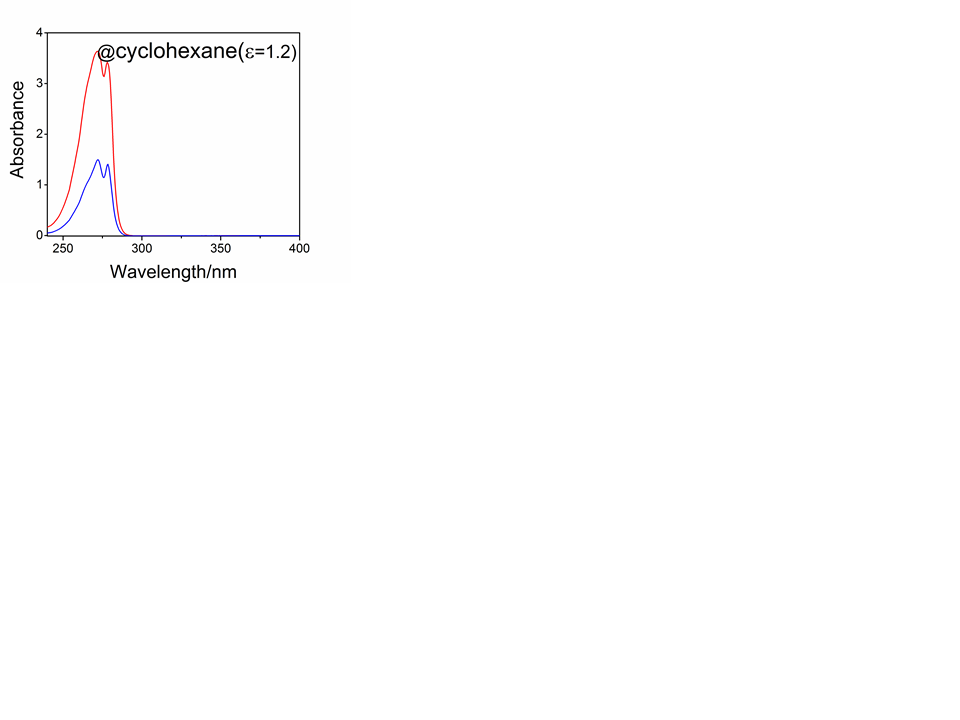


**Figure S1**. The UV/Vis absorption spectra of *o*-cresol in water, DMF, acetonitrile, ethanol, THF, ethyl acetate, chloroform, diethyl ether, toluene, benzene, CCl_4_, hexane, and cyclohexane at different concentrations (the cuvette with 10mm optical path length).

**Figure S2.** The UV/Vis absorption spectra of *o*-cresol in toluene, water, CCl_4_ and benzene at concentration of 5.6˟10^-4^ M using the cuvette with either 1mm (black line) or 10mm (red line) optical path length. The 1 mm path-length UV/Vis spectrum of *o*-cresol solvated in benzene shows the feature absorption bands of benzene in 240-270 nm (shown in reference: T. F. Kahan; D. J. Donaldson, *Environmental Science & Technology*, 2010, **44**, 3819-3824), which overlaps with the absorption of *o*-cresol at 270-280 nm.


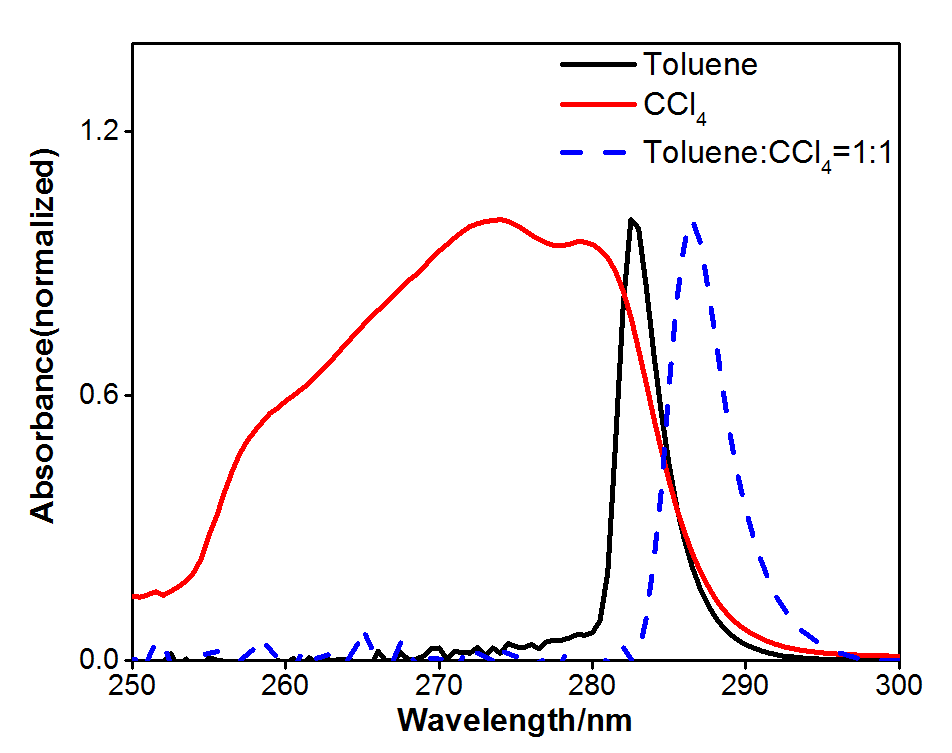


**Figure S3**. Normalized absorption spectra of *o*-cresol in toluene, CCl_4_, and the toluene/CCl_4_ mixture with volume ratio =1:1.

**S2. Experimental and Calculated IR Spectra.**

**S2.1 The Calculations for IR Spectra.**

In our calculations of IR spectra, frequency calculations were performed to mainly study typical stretching mode (with scale factor of 0.97) and to identify all the stationary points as local minima at the B3LYP/6-311+G(d,p) level.


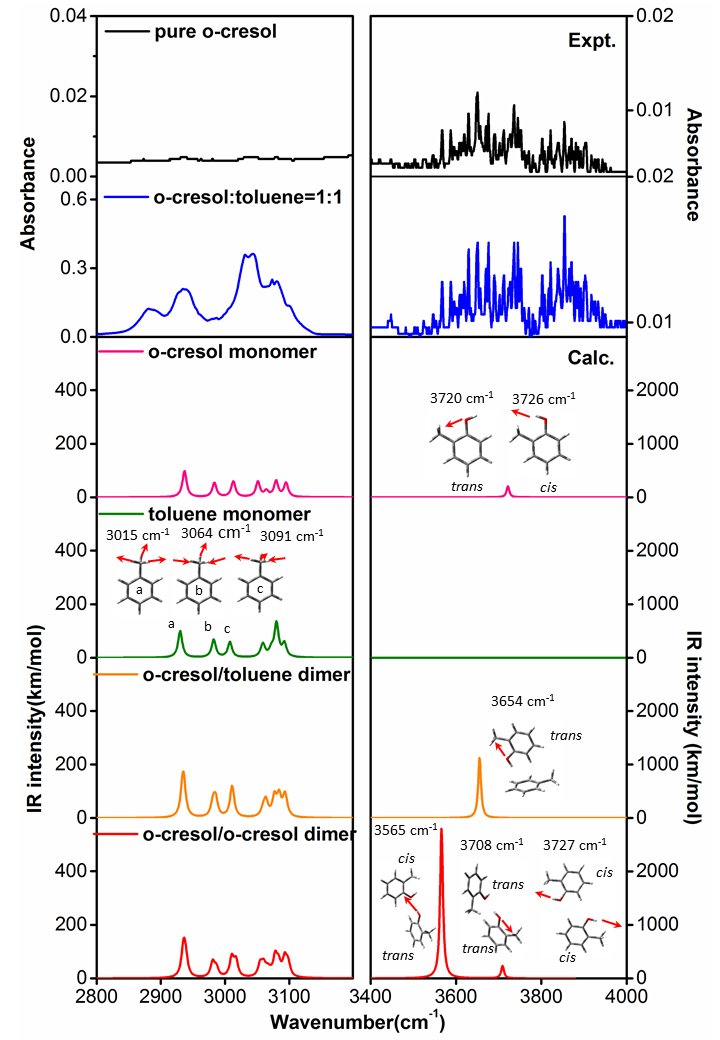


**Figure S4.** Experimental Infrared (IR) spectroscopy for pure o-cresol (vapor),

*o*-cresol/toluene mixture with volume ratio 1:1 (vapor) and the calculated IR spectra with scale factor of 0.97. The vibration modes at specific wavenumber of each monomer and dimers are also depicted inset.

**S3. Computational Details**

**S3.1 Detailed Information of MD Simulations.**

**S3.1.1 Simulation Models.**

**Table S1.**  Details of various simulation models used in NVT MD simulations with PCFF at 298 K.


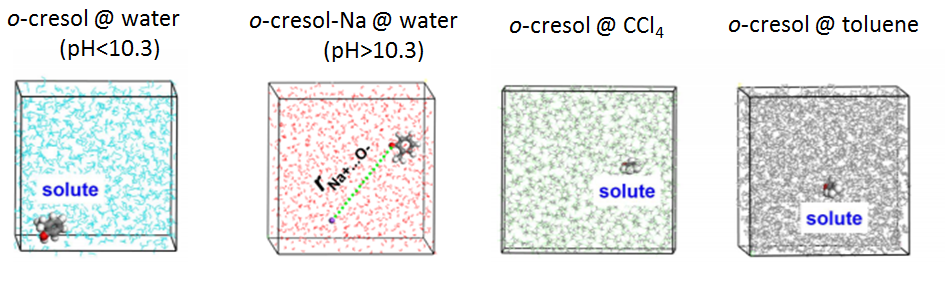


| substance | N_solute_ | N_solvent_ | C (mol/L) | ρ (g/cm3) | PBC cell/Å |
| --- | --- | --- | --- | --- | --- |
| o-cresol@H2O | 1 | 1000 | 0.06 | 1.00 | 31×31×31 |
| o-cresol-Na@H2O | 1 | 1000 | 0.06 | 1.00 | 31×31×31 |
| o-cresol@Toluene | 1 | 1000 | 0.09 | 0.86 | 56×56×56 |
| o-cresol@CCl4 | 1 | 1000 | 0.09 | 1.59 | 54×54×54 |

**Table S2**. The computational details of different types of theoretical calculations.

| Computational details | Geometry^a^  (opt, freq) | Excitation energy^a^  (20singlet states) | E_int_^b^ | NBO analysis^a^ |
| --- | --- | --- | --- | --- |
| *o*-cresol/toluene | M06-2X(Mp2) | CAM-B3LYP | M06-2X | CAM-B3LYP |
| *o*-cresol/H2O | M06-2X(B3LYP) | CAM-B3LYP | M06-2X | CAM-B3LYP |
| *o*-cresol/CCl4 | M06-2X(B3LYP) | CAM-B3LYP | M06-2X | CAM-B3LYP |
| *o*-cresol-anion/Na^+^ | M06-2X(B3LYP) | CAM-B3LYP | M06-2X | CAM-B3LYP |
| *o*-cresol/DMF | B3LYP | - | M06-2X | - |
| *o*-cresol/acetonitile | B3LYP | - | M06-2X | - |
| *o*-cresol/ethanol | B3LYP | - | M06-2X | - |
| *o*-cresol/THF | B3LYP | - | M06-2X | - |
| *o*-cresol/ethyl acetate | B3LYP | - | M06-2X | - |
| *o*-cresol/chloroform | B3LYP | - | M06-2X | - |
| *o*-cresol/diethyl ether | B3LYP | - | M06-2X | - |
| *o*-cresol/hexane | B3LYP | - | M06-2X | - |
| *o*-cresol/cyclohexane | B3LYP | - | M06-2X | - |

^a^The calculations were performed at 6-311+G(d,p) basis set, ^b^The calculations were calculated at 6-311++G(d,p) with basis set superposition error (BSSE) correction.

**S3.2 Details of Energy Decomposition.**

Symmetry-adapted perturbation theory (SAPT) provides a means of directly computing the noncovalent interaction between two molecules, that is, the interaction energy is determined without computing the total energy of the monomers or dimer. In addition, SAPT provides a decomposition of the interaction energy into physically meaningful components: *i.e.*, electrostatic, exchange, induction, and dispersion terms.

A standard zeroth-order SAPT calculation (SAPT(0)/jun-ccpVDZ) was performed on the MP2/6-311+G(d,p) optimized structures of the T-shaped and PD-stack dimers of o-cresol/toluene and o-cresol/benzene. Jun-cc-pVDZ corresponds to reduced ang-cc-pVDZ basis set (without diffuse functions on hydrogen and without diffuse d functions on heavy atoms).

The total SAPT interaction energy was then calculated as

$$E_{SAPT0}=E_{el}^{(1)}+E_{exch}^{(1)}+E_{ind}^{(2)}+E_{ind-exch}^{(20)}+E_{disp}^{(2)}+E_{disp-exch}^{(2)}+\delta(\mathrm{HF})$$

where E_el_^(1)^ is the first-order electrostatic interaction energy, E^(1)^_exch_ the first-order exchange contribution, E^(2)^_ind_ and E^(2)^_ind–exch_ the second-order induction energy and its accompanying exchange-correction, E^(2)^_disp_ and E^(2)^_disp–exch_ the second-order dispersion and exchange-dispersion contributions, and δ(HF) an estimate of higher-order induction and exchange-induction contributions. The latter was determined from the difference of supermolecular counterpoise-corrected Hartree–Fock and Hartree–Fock level SAPT calculations of the sum E_el_^(1)^ + E^(1)^_exch_ + E^(2)^_ind_ + E^(2)^_ind–exch_ + E^(2)^_disp_ with the aug-cc-pVTZ basis set. For the purpose of analysis it is convenient to group some of the above contributions to the total induction energy E_IND_ = E^(2)^_ind_ + E^(2)^_ind–exch_ + δ (HF) and to the total dispersion energy E_DISP_ = E^(2)^_disp_ + E^(2)^_disp–exch_.

Table S3.Energy decompositions (kcal/mol) from a SAPT(0)/jun-cc-pVDZ analysis for the MP2/6-311+G(d,p) optimized geometries of o-cresol/toluene and o-cresol/benzene dimers.

| species | isomer | E_int_ | E_el_^(1)^ | E^(1)^_exch_ | E_IND_ | E^(2)^_ind_ | E^(2)^_ind-exch_ | E_DISP_ | E^(2)^_disp_ | E^(2)^_disp-exch_ | δ (HF) |
| --- | --- | --- | --- | --- | --- | --- | --- | --- | --- | --- | --- |
| o-cresol/toluene | T-shaped | -1.15 | -3.90 | 3.59 | -2.46 | -1.95 | 1.13 | -3.71 | -4.06 | 0.35 | -1.64 |
|  | PD-stack | -1.29 | -7.27 | 17.70 | 7.86 | -8.61 | 7.98 | -13.64 | -16.27 | 2.63 | 8.49 |
| o-cresol/benzene | T-shaped | -1.17 | -5.66 | 9.51 | 0.01 | -4.15 | 2.81 | -6.39 | -7.32 | 0.93 | 1.35 |
|  | PD-stack | -1.18 | -6.37 | 15.80 | 7.09 | -7.52 | 6.95 | -12.19 | -14.54 | 2.35 | 7.66 |

Table S4 The distance from center of aromatic ring to center of another aromatic ring in PD-stack isomers, relative energy of isomers, intermolecular interaction in energies between different aromatic molecules, intermolecular orbital interaction energies between the bonding and antibonding orbitals, the maximum absorption peak and osilator strength of each dimers.

|  | R^a^(Å) | ΔE ^a^ (kcal/mol) | E_int_^b^  (kcal/mol) | E_orb_ ^c^ (kcal/mol) | λmax^c^ (nm)/f |
| --- | --- | --- | --- | --- | --- |
| ***o*-cresol/toluene** |  |  |  |  |  |
| T-shaped |  | 3.30 | -1.15 | 1.30(π→σ*/σ→π*) | 265/0.0516 |
| PD-stack | 3.57 | 0.00 | -1.29 | 2.20(π→π*) | 270/0.0316 |
| ***o*-cresol/benzene** |  |  |  |  |  |
| T-shaped |  | 0.44 | -1.17 | 3.14(π→σ*/n→π*) | 263/0.0358 |
| PD-stack | 3.56 | 0.00 | -1.18 | 0.90(π→π*) | 264/0.0241 |
| **Phenol/toluene** |  |  |  |  |  |
| T-shaped |  | 0.01 | -1.19 | 0.35(σ→π*) | 266/0.0367 |
| PD-stack | 3.59 | 0.00 | -1.16 | 2.59(π→π*) | 268/0.0181 |
| **Phenol/benzene** |  |  |  |  |  |
| T-shaped |  | 0.25 | -1.21 | 3.23(π→σ*) | 265/0.0351 |
| PD-stack | 3.63 | 0.00 | -1.03 | 1.17(π→π*) | 267/0.0170 |
| **Benezene/benzene** |  |  |  |  |  |
| T-shaped |  | 0.41 | -0.54 | 0.49(π→σ*) | 251/0.0001 |
| PD-stack | 3.66 | 0.00 | -0.64 | 1.20(π→π*) | 254/0.0001 |
| **Benzene/toluene** |  |  |  |  |  |
| T-shaped |  | 0.03 | -0.42 | 0.23(π→σ*) | 256/0.0019 |
| PD-stack | 3.62 | 0.00 | -0.85 | 1.49(π→π*) | 260/0.0010 |

^a^The calculations were performed at MP2/6-311+G(d,p) level. ^b^The calculations were performed at M06-2X/6-311+G(d,p) level. ^c^The calculations were performed at CAM-B3LYP/6-311+G(d,p) level.

Table S5 The NBO charges in different solute/solvent dimers,

|  | NBO charge (e)^a^ | | Δ*q_CT_*  (*q*_solute_-*q*_solvent_) | NBO charge (e)^a^ | | | Δ*q*  (*q*_O_-*q*_H_) |
| --- | --- | --- | --- | --- | --- | --- | --- |
|  | *q*_solute_ | *q*_solvent_ |  | *q*_H_ | | *q*_O_ |  |
| **@toluene** |  |  |  |  |  | |  |
| T-shaped | -0.003 | 0.003 | -0.006 | -0.71 | 0.49 | | 1.20 |
| PD-stack | 0.004 | -0.004 | -0.008 | -0.70 | 0.48 | | 1.18 |
| **@benzene** |  |  |  |  |  | |  |
| T-shaped | -0.006 | 0.006 | -0.012 | -0.70 | 0.49 | | 1.19 |
| PD-stack | 0.005 | -0.005 | 0.010 | -0.69 | 0.48 | | 1.17 |
| **@CCl_4_** |  |  |  |  |  | |  |
| C-Cl…π *trans/cis* | 0.002/0.002 | -0.002/-0.002 | 0.004/0.004 | -0.70/-0.70 | 0.48/0.48 | | 1.18/1.18 |
| O-H…Cl/C-Cl…O *trans/cis* | -0.003/0.002 | 0.003/-0.002 | -0.006/0.004 | -0.70/-0.70 | 0.48/0.48 | | 1.18/1.18 |
| **@H_2_O** **pH<10.3** |  |  |  |  |  | |  |
| *trans/cis* | 0.012/0.012 | -0.012/-0.012 | 0.024/0.024 | -0.73/-0.73 | 0.50/0.50 | | 1.23/1.23 |
| **@H_2_O** **pH>10.3** |  |  |  |  |  | |  |
| ion pair | - | - | - | - | -1.03 | | - |
| cation…π | - | - | - | - | -0.73 | | - |


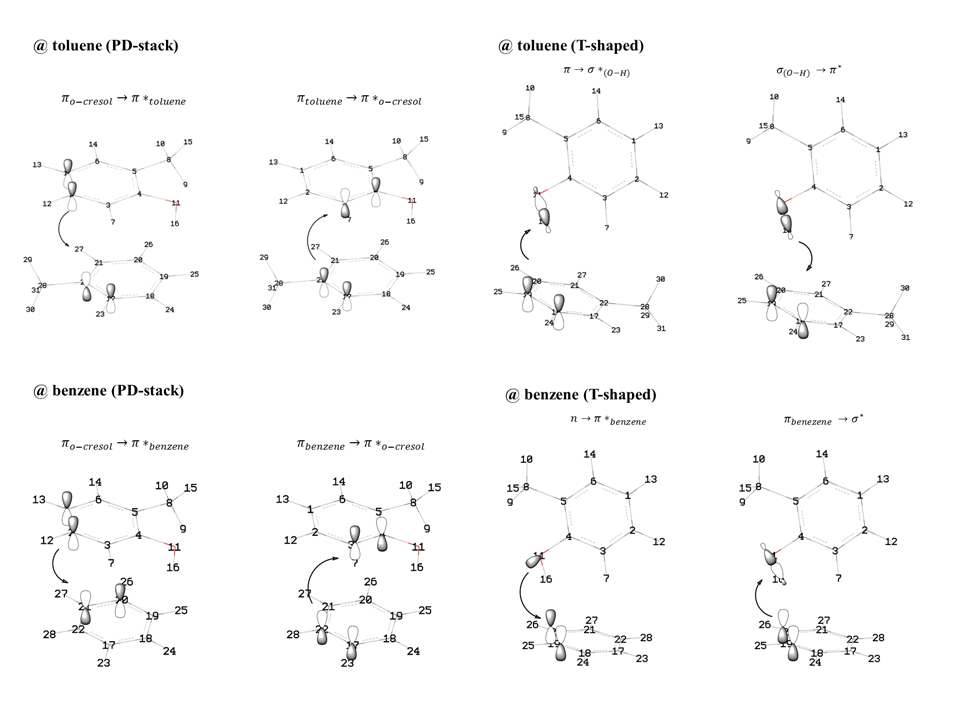

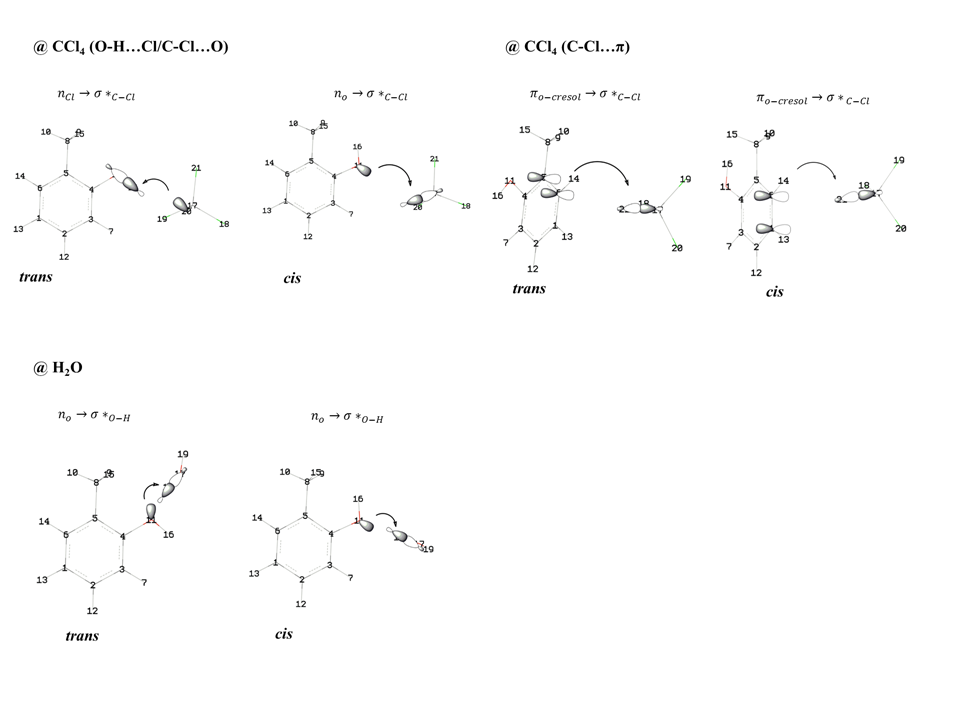


Figure S5. NBO analysis of the calculation (CAM-B3LYP/6-311+G(d,p)) of the *o*-cresol/toluene, *o*-cresol/benzene, *o*-cresol/CCl_4_, and *o*-cresol/H_2_O dimers.

**Figure S6**. (a) MP2/6-311+G(d,p) optimized T-shaped (T) and parallel-displaced (PD) structures of the o-cresol/toluene complex. (b) The structures of o-cresol/H2O (pH<10.3) and o-cresol anion…Na+ (pH>10.3) computed at B3LYP level in vacuum. (c) the optimized structures of CCl4/o-cresol complexes computed at B3LYP level. The the numbers closed to the bond are the bond orders (B.O.) from NBO analysis.

**Figure S7. to be continued.**

**Figure S7.** The absorption spectrum of o-cresol/toluene dimer (a) was displayed in both column and curve forms at CAM-B3LYP/6-311+G(d,p) level in vacuum and the dominant orbital contributions for the maximum absorption peak. The frontier molecular orbitals of o-cresol/benzene dimer (b), o-cresol/CCl_4_(c).

**S3.3 Simulated UV/Vis Spectra in Different Solutions.**

**S3.3.1 Cluster Model for Simulating UV/Vis Spectra in Solutions**.

The specific interactions may be modeled by means of cluster model made of an aggregate of the solute and a limited number of solvent molecules treated as an isolated single molecular system at the desired quantum chemical level. This approach may give useful information on intermolecular interactions. The clusters were extracted from the MD simulations with different distances (4 Å, 6 Å, 7 Å, 8 Å) from the center of solute molecule. Then the lowest 20 excitation energies were calculated at CAM-B3LYP/6-311+G(d,p) level.

**Figure S8.** The radial distribution function, *g*(r), for the r _O…C_ distance between o-cresol oxygen atom (O) and the centroid of aromatic ring in toluene (a) and carbon atom of CCl_4_ (b).


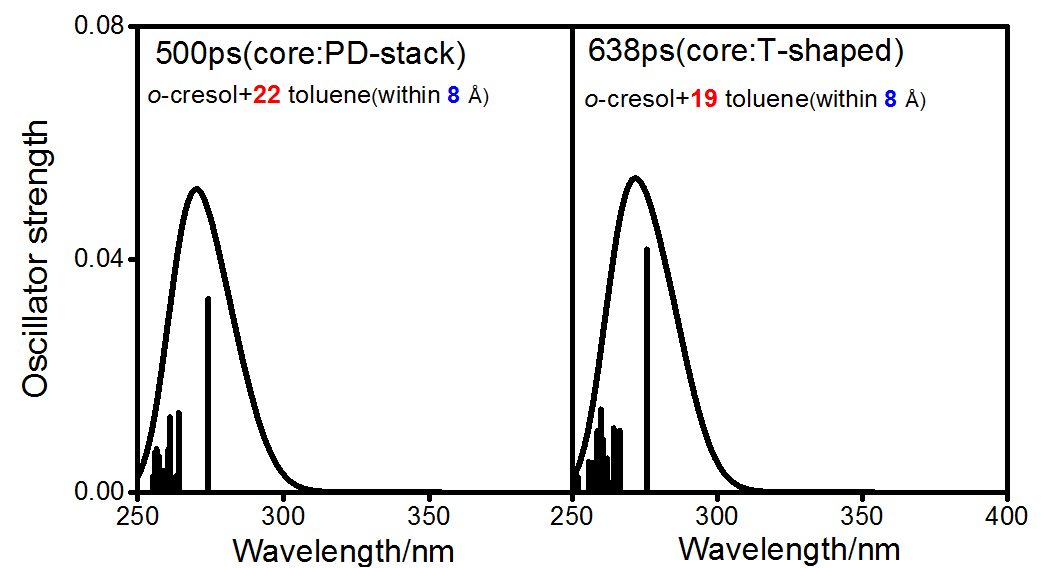


**Figure S9.** The calculated spectra (with scale factor of 1.1) based on the captured snapshots at 500 ps and 638 ps with the core conformation of PD-stack and T-shaped, respectively. The calculated absorption spectrum (line) is smoothed by a Gaussian function with a width of 0.2 eV at half height.


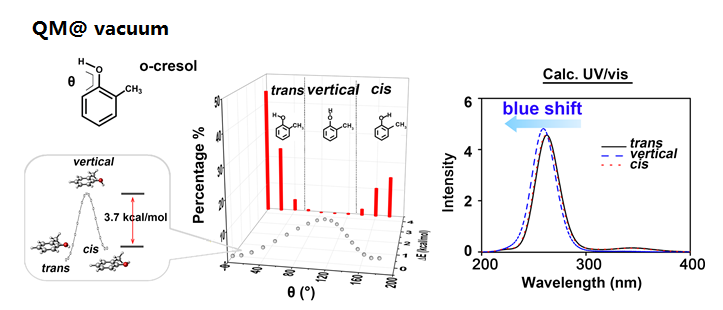


**Figure S10.**  The percentage of different isomers of o-cresol conformations according to the Boltzmann distribution from rotational energy curve in vacuum and the calculated absorption spectra of the isomers in vacuum.

**S3.3.2 The combined MD and TDDFT approach for Simulating UV/Vis Spectra in Solutions**.

Two independent MD simulations were carried out with either *trans* or *cis* initial structures of *o*-cresol in toluene and aqueous solutions. From the simulated 1ns MD trajectories, the snapshots were progressively captured from the 200ps to 1000ps, with Δt=25 ps, A total of 33 snapshots were introduced to simulate the spectra in solutions. The solvent shells were extracted with 4 Å and 6 Å from the center of *o*-cresol in toluene and aqueous solutions, respectively. The lowest 20 excitation energies were calculated at CAM-B3LYP/6-311+G(d,p). The spectra were obtained using a Gaussian line broadening which was embedded in GaussView5.0.8:

$\varepsilon(\lambda) = \sum_{i} \varepsilon_{i}(\lambda)$ (2)

$\varepsilon_{i}(\lambda)=\frac{\sqrt{\pi}e^{2}N}{1000\ln(10)c^{2}m_{e}}\cdot\frac{f_{i}}{\sigma}\exp[-(\frac{hc/\lambda-hc/\lambda_{i}}{hc/\sigma_{\lambda}})^{2}]$ (3)

where *i* indicates the excited state, e is the charge of the electron (4.803204*10^-10^ esu), *m_e_* is the electron mass (9.10938 *10^-31^ g), *N* is Avogadro’s number (6.02214199*10^23^), *h* is Planck’s constant (4.135668*10^15^ eV s) and c is the speed of light (299792458.0 m s^-1^). λ_i_ and *f*_i_ are the excitation energy (in nm) and oscillator strength (dimensionless) of excitation i, respectively. This procedure leads to UV/Vis spectra in units of L mol^-1^ cm^-1^. The standard deviation was chosen to be σ = 0.053 eV(425cm^-1^)


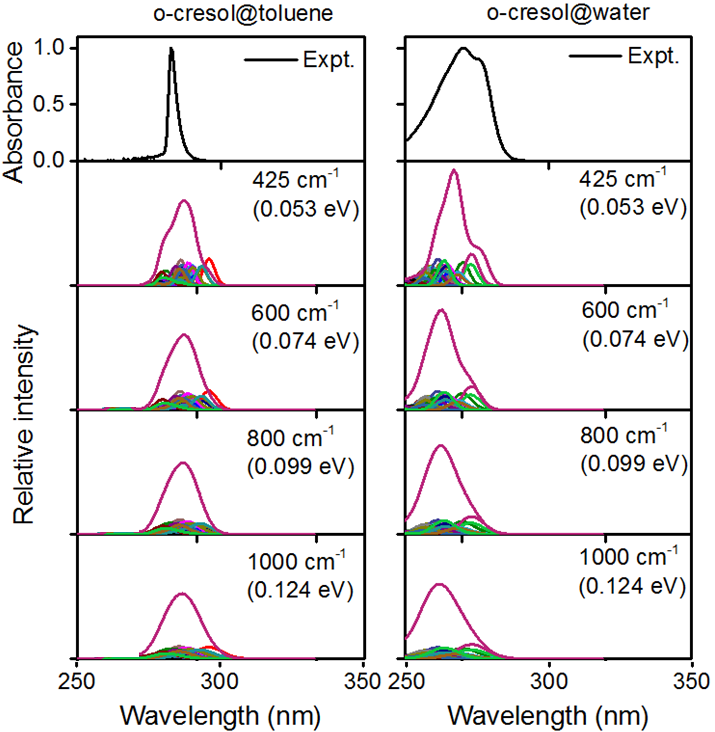


**Figure S11.** The experimental spectra in toluene and in aqueous solutions, and statistic average of the calculated spectra (scale factor=1.1) sampled at 1ns-simulation in solutions with different broaden width. (425 cm^-1^, 600 cm^-1^, 800 cm^-1^, 1000 cm^-1^).

**Figure S12.** The distribution of torsion angle C-C-O-H of *o*-cresol in water and toluene solvents, respectively.


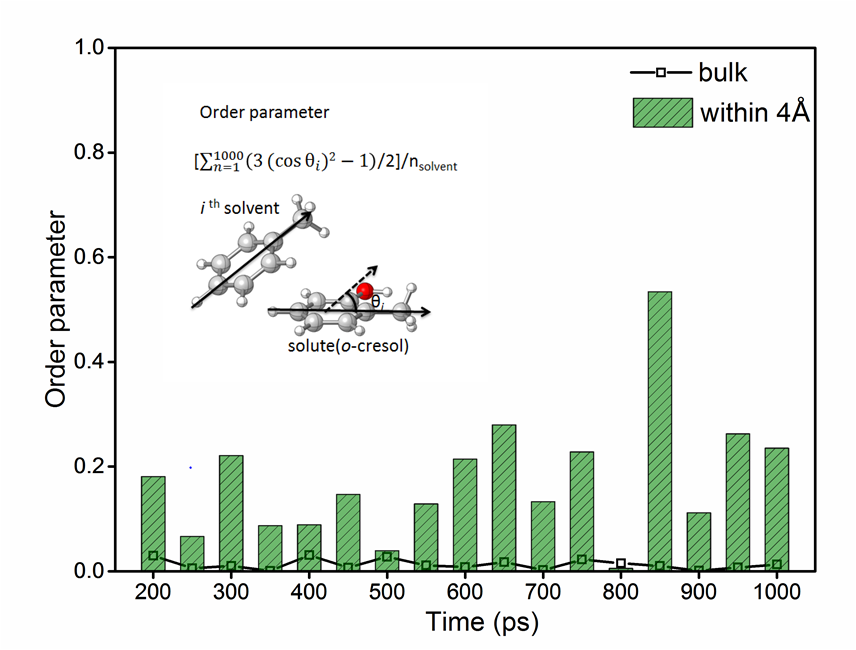


**Figure S13.** The order parameter defined in toluene solutions, and the difference between bulk solution and clusters within 4A from solute *o*-cresol.

**Figure S14.** The absorption spectrum of o-cresol anion /Na^+^ ion pair (a) and different o-cresol anion/water cluster model (b) were displayed in both column and curve forms at CAM-B3LYP/6-311+G(d,p) level and the dominant orbital contributions for the maximum absorption peak. In the coloured isosurface plot, green colour denotes weak H-bond and red colour stands for steric effect. The orbital analyses for the maximum absorption wavelength are shown in inset.

**
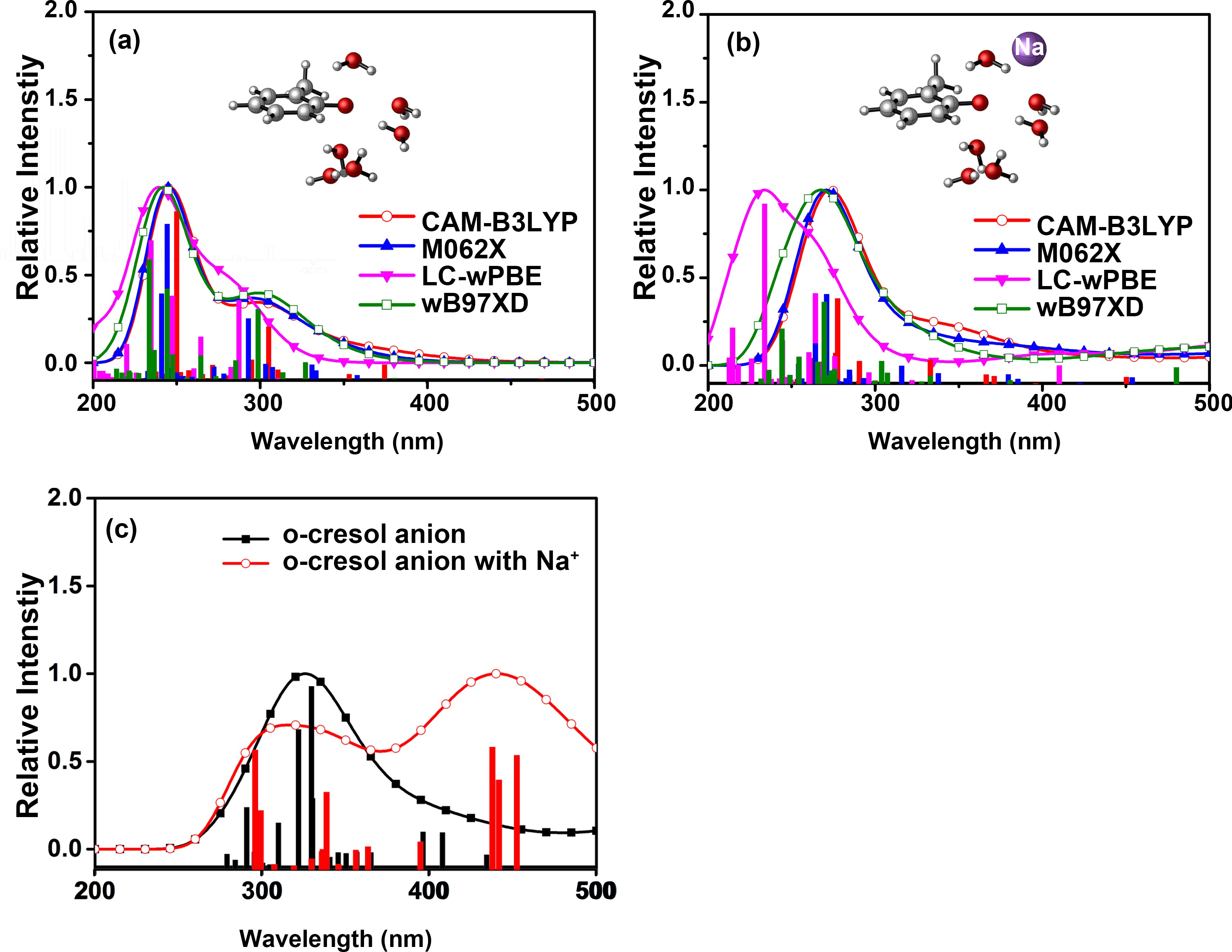
**

**Figure S15.** The UV/Vis absorption spectra of (a) the *o*-cresol anion with 6H_2_O at 2 ps, (b) its corresponding Na salt using different functionals.

**
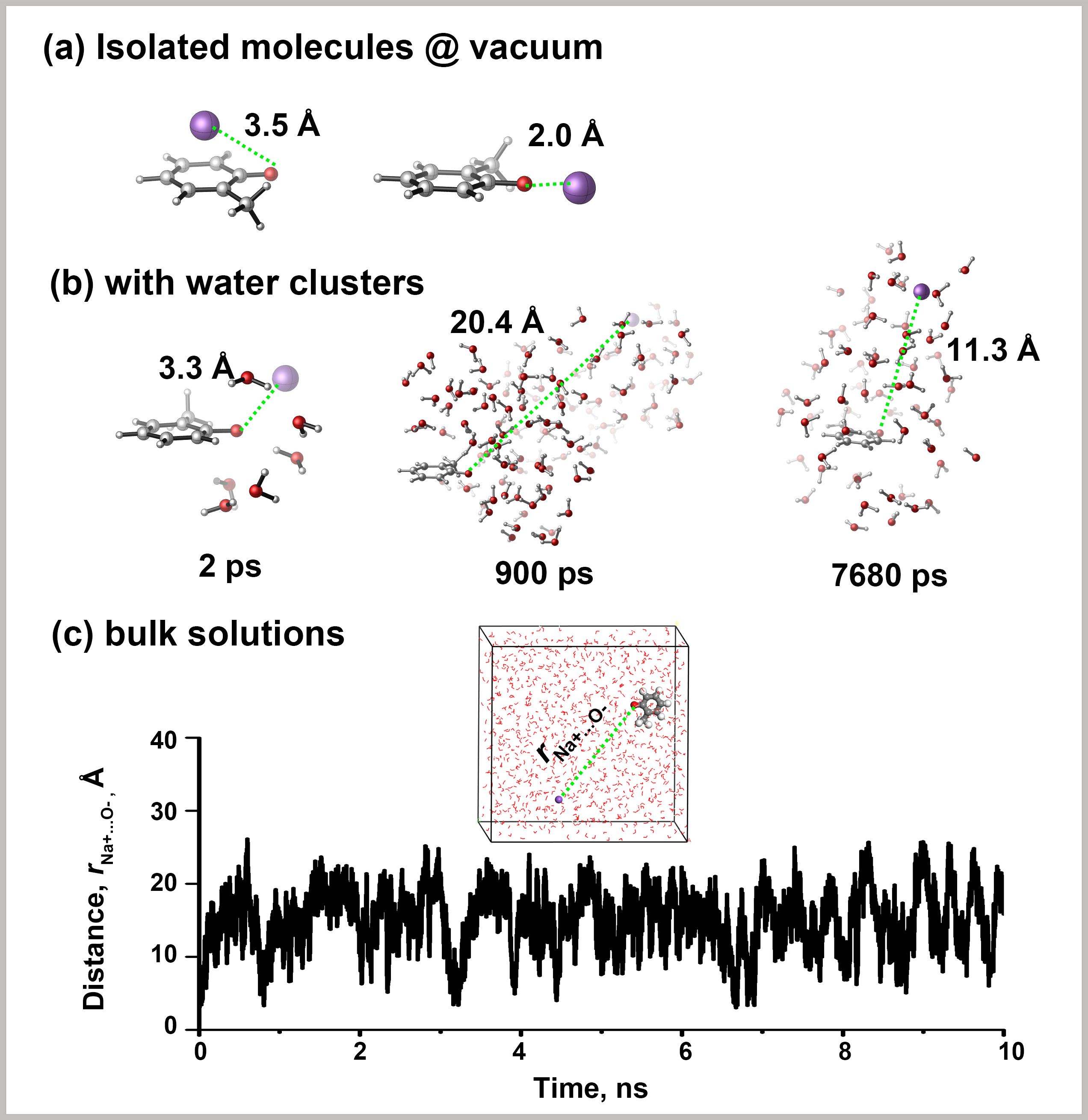
**

**Figure S16.** The structures of *o*-cresol anion…Na^+^ (a) in vacuum, (b) with water clusters, and the distance of Na^+^ cation and O atom of the *o*-cresol anion in 10ns MD simulation trajectory

S4. Cartesian coordinates of of all involved molecular structures.

**o-cresol/toluene(PD-stack,MP2 level)**

C 1.277115 2.328938 -0.123547

C 0.512215 1.998742 1.006545

C 0.542528 0.689774 1.515548

C 1.336292 -0.288435 0.894409

C 2.121177 0.027206 -0.237278

C 2.077581 1.343385 -0.729569

H -0.050200 0.424619 2.399270

C 2.963717 -1.050491 -0.870213

H 2.337753 -1.912386 -1.157702

H 3.481412 -0.670876 -1.765359

O 1.408376 -1.581681 1.348261

H -0.105799 2.756818 1.499640

H 1.262342 3.348238 -0.523460

H 2.681794 1.595690 -1.609303

H 3.719303 -1.429819 -0.161307

H 0.644775 -1.715321 1.930261

C -2.503509 -0.529721 0.598739

C -1.958702 -1.825046 0.537639

C -1.058658 -2.163150 -0.488180

C -0.707125 -1.194918 -1.445091

C -1.257415 0.095521 -1.379973

C -2.158825 0.448046 -0.356189

H -3.205004 -0.272614 1.402079

H -2.248477 -2.574069 1.284167

H -0.626269 -3.167901 -0.535890

H -0.004981 -1.446540 -2.247359

H -0.966904 0.847056 -2.123137

C -2.759101 1.833887 -0.305410

H -2.028359 2.588035 -0.640298

H -3.645687 1.906421 -0.960243

H -3.076452 2.093674 0.717978

**o-cresol/toluene(T-shaped,MP2 level)**

C -3.305249 1.928326 0.246603

C -1.924429 2.024146 0.390733

C -1.129623 0.886052 0.263531

C -1.718897 -0.348792 -0.008222

C -3.110594 -0.469893 -0.157302

C -3.880639 0.686133 -0.024717

H -0.051683 0.951374 0.376812

C -3.725219 -1.813515 -0.449606

H -3.492595 -2.535771 0.338617

H -4.810638 -1.733012 -0.534480

O -0.981276 -1.495115 -0.141494

H -1.459747 2.980575 0.603112

H -3.930414 2.808115 0.344328

H -4.957495 0.607863 -0.136825

H -3.335825 -2.233432 -1.381928

H -0.041301 -1.303642 -0.025141

C 2.987824 0.536691 1.029364

C 2.641471 -0.697757 1.577695

C 2.386017 -1.787055 0.745305

C 2.480453 -1.628301 -0.639035

C 2.825229 -0.390779 -1.180556

C 3.083579 0.711181 -0.356103

H 3.189619 1.376063 1.687181

H 2.574801 -0.809690 2.654116

H 2.125816 -2.750541 1.168831

H 2.291508 -2.470052 -1.296240

H 2.897538 -0.280116 -2.257856

C 3.436480 2.053185 -0.950903

H 4.126552 1.945917 -1.791672

H 2.542951 2.562869 -1.326871

H 3.902708 2.706670 -0.210751

o-cresol/toluene(PD-stack ,M06-2X level)

C 1.231157 2.237941 0.331454

C 0.489001 1.607765 1.321806

C 0.623692 0.239656 1.527696

C 1.494232 -0.495845 0.733025

C 2.257660 0.116549 -0.266690

C 2.108513 1.487598 -0.449444

H 0.033118 -0.263575 2.286848

C 3.180632 -0.719295 -1.108256

H 2.631129 -1.526819 -1.598734

H 3.667410 -0.110098 -1.870258

O 1.643022 -1.845836 0.878055

H -0.204526 2.174557 1.932643

H 1.132571 3.304105 0.165853

H 2.689946 1.974910 -1.225604

H 3.950341 -1.191847 -0.493257

H 0.924706 -2.182851 1.421705

C -2.722589 -0.229150 0.515790

C -2.365535 -1.572969 0.613565

C -1.442362 -2.115004 -0.273279

C -0.877270 -1.303639 -1.255480

C -1.235639 0.033884 -1.346522

C -2.166101 0.589639 -0.464148

H -3.442015 0.187886 1.213370

H -2.815196 -2.194988 1.379641

H -1.165667 -3.161419 -0.207407

H -0.143275 -1.714271 -1.939974

H -0.772669 0.666532 -2.097526

C -2.534279 2.045830 -0.574386

H -1.633985 2.665020 -0.587252

H -3.084903 2.240019 -1.498525

H -3.157837 2.361772 0.263253

o-cresol/toluene(T-shaped ,M06-2X level)

C -2.329369 1.983124 0.181497

C -1.122623 1.883941 0.863710

C -0.587059 0.633179 1.151694

C -1.255909 -0.515166 0.743809

C -2.475479 -0.440784 0.060518

C -2.993965 0.822047 -0.208561

H 0.367590 0.544317 1.663346

C -3.163377 -1.707055 -0.365420

H -3.411015 -2.326227 0.500140

H -4.081128 -1.483468 -0.909915

O -0.758029 -1.763692 0.978837

H -0.590313 2.776291 1.171886

H -2.753041 2.953962 -0.044184

H -3.936876 0.894452 -0.740733

H -2.510588 -2.306508 -1.004914

H 0.158123 -1.686487 1.267467

C 2.150558 1.173767 -0.662378

C 3.114139 1.081099 0.333946

C 3.450194 -0.160642 0.869305

C 2.828478 -1.305044 0.381595

C 1.865643 -1.205144 -0.622408

C 1.501281 0.034933 -1.145856

H 1.874339 2.146262 -1.057996

H 3.599466 1.978382 0.700412

H 4.197690 -0.236315 1.650047

H 3.098344 -2.279168 0.774811

H 1.378904 -2.101248 -0.993716

C 0.403985 0.161670 -2.168762

H -0.051381 -0.807557 -2.378364

H -0.380030 0.829549 -1.798412

H 0.785166 0.573755 -3.106700

Trans o-cresol/CCl4(O-H…Cl/M06-2X level)

C 5.509256 -0.485197 -0.195436

C 5.355148 0.893821 -0.161233

C 4.087463 1.446792 -0.006943

C 2.981057 0.615015 0.112958

C 3.110178 -0.777048 0.080533

C 4.388667 -1.303498 -0.074057

H 3.957509 2.524501 0.020943

C 1.889002 -1.642935 0.210520

H 1.201358 -1.476005 -0.622412

H 2.165656 -2.697391 0.226705

O 1.714073 1.110944 0.268772

H 6.215230 1.546016 -0.252719

H 6.492243 -0.923654 -0.314558

H 4.505301 -2.381896 -0.098530

H 1.340208 -1.409359 1.126061

H 1.741334 2.071773 0.282646

C -2.768297 0.020636 -0.030112

Cl -3.296904 -0.820564 1.441961

Cl -3.039156 -1.030408 -1.436436

Cl -3.713030 1.512487 -0.224304

Cl -1.047748 0.412188 0.093150

Trans o-cresol/CCl4 (C-Cl…pi/M06-2X level)

C 2.301959 2.030437 -0.443851

C 1.678469 1.920447 0.791012

C 1.563442 0.675502 1.402993

C 2.074599 -0.454545 0.774666

C 2.689200 -0.370699 -0.479947

C 2.796125 0.885600 -1.067008

H 1.073108 0.580331 2.367791

C 3.191997 -1.622738 -1.140497

H 2.374792 -2.333605 -1.287621

H 3.639146 -1.396313 -2.108650

O 2.004762 -1.696073 1.337386

H 1.274643 2.797330 1.282828

H 2.398020 2.996101 -0.924629

H 3.276361 0.966244 -2.036957

H 3.937109 -2.121524 -0.516432

H 1.548051 -1.641667 2.181266

C -1.798596 -0.059207 -0.122328

Cl -1.235548 -1.509167 0.739314

Cl -3.374993 -0.399655 -0.864730

Cl -1.971779 1.282031 1.029119

Cl -0.626063 0.373433 -1.371169

Cis o-cresol/CCl4 (C-Cl…O/M06-2X level)

-4.788976 -1.427785 -0.011155

-3.523370 -2.012210 0.003798

-2.385113 -1.213359 0.015298

-2.511140 0.175354 0.012192

-3.774345 0.788289 -0.002702

-4.900649 -0.039579 -0.014175

-1.391712 -1.645175 0.026946

-3.900606 2.292450 -0.005752

-3.428598 2.744041 -0.887180

-4.949461 2.592119 -0.016364

-1.347591 0.902019 0.024233

-3.419530 -3.091394 0.006369

-5.679497 -2.045014 -0.020301

-5.883996 0.419694 -0.025708

-3.446041 2.746061 0.883769

-1.545293 1.843753 0.020475

2.633703 -0.030851 -0.002203

4.379323 -0.447630 -0.020652

1.842352 -0.724830 -1.448687

1.879912 -0.696561 1.477307

2.452993 1.752897 -0.017008

Cis o-cresol/CCl4 (C-Cl…pi/M06-2X)

C 2.752403 1.822915 -0.966153

C 3.132880 2.020446 0.360671

C 3.404331 0.929451 1.178771

C 3.295267 -0.364915 0.671308

C 2.913433 -0.591115 -0.660786

C 2.648024 0.524624 -1.460962

H 3.700111 1.058483 2.213038

C 2.798988 -1.995207 -1.202350

H 2.053302 -2.587739 -0.657959

H 2.492747 -1.981433 -2.249111

O 3.577562 -1.392410 1.534427

H 3.217923 3.024200 0.761264

H 2.540231 2.668645 -1.609393

H 2.354626 0.365722 -2.493874

H 3.753410 -2.533695 -1.152510

H 3.456504 -2.238552 1.092313

C -2.398845 -0.007448 0.057457

Cl -2.405634 -1.083693 1.492289

Cl -3.199583 -0.844099 -1.314153

Cl -3.296065 1.496708 0.439091

Cl -0.714497 0.391739 -0.384692

Cis o-cresol/H2O (M06-2X)

C -1.83208900 -0.90217300 0.00004300

C -1.99841800 0.48196600 -0.00007300

C -0.88749800 1.31842800 -0.00006500

C 0.39471600 0.77067500 -0.00006000

C 0.58892300 -0.61989200 -0.00001400

C -0.54581600 -1.43627700 0.00013000

H -0.99173700 2.39688100 0.00003100

C 1.98090600 -1.20390100 -0.00012900

H 2.55443200 -0.90308400 0.88551600

H 1.94182400 -2.29402100 -0.00058400

O 1.44448200 1.65422700 0.00008800

H -2.99306800 0.91324300 -0.00010300

H -2.69357100 -1.55939100 0.00000400

H -0.41172200 -2.51338900 0.00034100

H 2.55451300 -0.90238800 -0.88534300

H 2.27912900 1.17538500 0.00044300

O 2.13700041 4.15098039 0.01832237

H 1.92542542 3.22069570 0.12512319

H 1.33081187 4.64645107 -0.14344550

Trans-o-cresol/H2O(M06-2X)

C -2.62063500 0.29599300 0.36733300

C -2.34130000 -1.05934500 0.22254000

C -1.04930600 -1.46905600 -0.10083900

C -0.04564400 -0.52079700 -0.27914400

C -0.29881600 0.85064700 -0.14048400

C -1.60231500 1.23131800 0.18495300

H -0.82125600 -2.52538200 -0.21223800

C 0.80207900 1.86032400 -0.33679200

H 1.27548800 1.74597200 -1.31638300

H 0.40989500 2.87597100 -0.26312400

O 1.24600000 -0.89333500 -0.60242300

H -3.11988800 -1.80045000 0.36106700

H -3.62168200 0.62517600 0.61935700

H -1.82007400 2.28855900 0.29594100

H 1.58826400 1.74562800 0.41597300

H 1.29047000 -1.85085700 -0.70213800

O 3.80811200 -0.35157000 0.71680800

H 2.94713500 -0.40590800 0.27705900

H 4.37437100 0.12602900 0.10400700

o-cresol-anion/Na+（ion pair/M06-2X）

C 2.651039 -0.259083 -0.000098

C 1.965083 -1.473621 0.000043

C 0.574226 -1.496754 0.000141

C -0.189309 -0.306137 0.000046

C 0.518922 0.931019 0.000101

C 1.913132 0.926814 -0.000026

H 0.038128 -2.441396 0.000218

C -0.261392 2.219086 0.000071

H -0.913911 2.291947 0.878500

H 0.406403 3.084526 0.000692

O -1.502144 -0.330729 -0.000240

H 2.516500 -2.409339 0.000026

H 3.734826 -0.232704 -0.000275

H 2.437280 1.879498 -0.000133

H -0.912978 2.292510 -0.879011

Na -3.483573 -0.460651 0.000022

o-cresol-anion/Na+(cation ….pi/M06-2X)

C -1.582982 -1.011200 -0.571553

C -1.812146 0.371666 -0.665167

C -0.775565 1.289611 -0.496419

C 0.602379 0.880146 -0.275170

C 0.811374 -0.573090 -0.200898

C -0.258013 -1.451833 -0.360024

H -0.957903 2.354716 -0.596592

C 2.228095 -1.054907 -0.042048

H 2.718287 -0.585817 0.816729

H 2.274186 -2.142025 0.064792

O 1.544660 1.693395 -0.118810

H -2.814482 0.734635 -0.877433

H -2.383516 -1.723135 -0.730220

H -0.059865 -2.521515 -0.334694

H 2.827229 -0.764290 -0.911514

Na -0.840006 0.036173 1.744281

o-cresol@toluene cluster(within 8Å) 500ps

C 25.55800000 41.56500000 17.08600000

C 26.21000000 41.77800000 15.88500000

C 27.46200000 41.26900000 15.68800000

C 28.24000000 40.82300000 16.75600000

C 27.55200000 40.58300000 17.94900000

C 26.29500000 41.10300000 18.19300000

H 24.54300000 41.84900000 17.13200000

H 25.69000000 42.13100000 14.93100000

H 27.88800000 41.15500000 14.69400000

H 28.11700000 40.13800000 18.77000000

H 25.72000000 40.70700000 19.08300000

C 29.72100000 40.56800000 16.68400000

H 30.18600000 41.13100000 15.83000000

H 30.13800000 40.99000000 17.60900000

H 30.05600000 39.53600000 16.53400000

C 30.82300000 50.94700000 0.11600000

C 32.11200000 50.73800000 0.54900000

C 32.58500000 49.45300000 0.72100000

C 31.88400000 48.36300000 0.20600000

C 30.53600000 48.65000000 -0.29700000

C 30.00200000 49.87700000 -0.24700000

H 30.39200000 51.91900000 -0.17500000

H 32.76900000 51.56000000 0.84600000

H 33.51300000 49.24100000 1.27000000

H 29.79500000 47.81100000 -0.55700000

H 28.98400000 50.06000000 -0.61300000

C 32.26800000 46.94500000 0.41100000

H 33.40900000 46.81200000 0.48000000

H 31.76900000 46.57200000 1.30400000

H 31.92400000 46.30900000 -0.37200000

C 25.80700000 47.37800000 16.00600000

C 25.18100000 47.74100000 14.83400000

C 23.94800000 48.38900000 14.91600000

C 23.46200000 48.82600000 16.14200000

C 24.15300000 48.65100000 17.27000000

C 25.38100000 47.90200000 17.25400000

H 26.76000000 46.89100000 15.84200000

H 25.48500000 47.25100000 13.88300000

H 23.31300000 48.52200000 14.05300000

H 23.80100000 49.21600000 18.23300000

H 25.78500000 47.64700000 18.20500000

C 22.15200000 49.54100000 16.27600000

H 21.21800000 48.91800000 16.12500000

H 22.06300000 49.90700000 17.28500000

H 22.16600000 50.39600000 15.61500000

C 33.96600000 34.65700000 17.81500000

C 32.71400000 34.97300000 17.24100000

C 32.19900000 36.27100000 17.39500000

C 32.82000000 37.20800000 18.22000000

C 34.02400000 36.89300000 18.79200000

C 34.57000000 35.63000000 18.57800000

H 34.37200000 33.64200000 17.76000000

H 32.27200000 34.20000000 16.56200000

H 31.24200000 36.44100000 16.83700000

H 34.62100000 37.60800000 19.37700000

H 35.55400000 35.50900000 19.02600000

C 32.17200000 38.54700000 18.61500000

H 31.28900000 38.68300000 17.96700000

H 31.72400000 38.60900000 19.63800000

H 32.81200000 39.42300000 18.42900000

C 27.40400000 33.94700000 1.74200000

C 26.66900000 33.79400000 2.93900000

C 26.73400000 34.85800000 3.86700000

C 27.51000000 36.03500000 3.58600000

C 28.12300000 36.17300000 2.36400000

C 27.98700000 35.14800000 1.42600000

H 27.33300000 33.16500000 1.01500000

H 26.06000000 32.83100000 3.18500000

H 26.22200000 34.74500000 4.85400000

H 28.68800000 37.08600000 1.99000000

H 28.42100000 35.29100000 0.47200000

C 27.64200000 37.22100000 4.54700000

H 28.44300000 37.96500000 4.31600000

H 26.63900000 37.67100000 4.70700000

H 27.89100000 36.84100000 5.54700000

C 24.02500000 37.85400000 13.10200000

C 23.74700000 38.96000000 12.23600000

C 24.73800000 39.77200000 11.76700000

C 26.06600000 39.64300000 12.23200000

C 26.31400000 38.51300000 13.03700000

C 25.36700000 37.55800000 13.40200000

H 23.24300000 37.20800000 13.33700000

H 22.82600000 39.07100000 11.72200000

H 24.42000000 40.56100000 11.12300000

H 27.38200000 38.30100000 13.26500000

H 25.61200000 36.66900000 13.99200000

C 27.20500000 40.52600000 11.77400000

H 28.16100000 39.88400000 11.73200000

H 27.18800000 40.88500000 10.73400000

H 27.34800000 41.37000000 12.48900000

C 28.77000000 44.52700000 12.78500000

C 29.30700000 45.35700000 13.74900000

C 29.86300000 46.55100000 13.30800000

C 29.64100000 47.01400000 11.98600000

C 29.11900000 46.15000000 10.93500000

C 28.60800000 44.93900000 11.44200000

H 28.56900000 43.48100000 13.01500000

H 29.26800000 45.12400000 14.82400000

H 30.31500000 47.17300000 14.04500000

H 28.87300000 46.50900000 9.93000000

H 28.40200000 44.10900000 10.76500000

C 30.06900000 48.40900000 11.64700000

H 30.51700000 49.09200000 12.31100000

H 30.81500000 48.41100000 10.80400000

H 29.10700000 48.92200000 11.28000000

C 17.74300000 40.52300000 8.87700000

C 18.55400000 40.70000000 10.02000000

C 19.96300000 40.51300000 9.84100000

C 20.48900000 40.28200000 8.56300000

C 19.61800000 40.20700000 7.50700000

C 18.26300000 40.27700000 7.60000000

H 16.63100000 40.79200000 8.93300000

H 18.09000000 40.88000000 11.03200000

H 20.58300000 40.51500000 10.77600000

H 20.08200000 40.01800000 6.53400000

H 17.59700000 40.27600000 6.64900000

C 22.03600000 40.22100000 8.39000000

H 22.38400000 39.59700000 7.51200000

H 22.44800000 41.28400000 8.30800000

H 22.48000000 39.74700000 9.25200000

C 33.14800000 47.54800000 14.77400000

C 33.16600000 48.67600000 15.55300000

C 33.24700000 49.99100000 14.95500000

C 33.21800000 50.10600000 13.54500000

C 33.36900000 48.94700000 12.76900000

C 33.28800000 47.71900000 13.39000000

H 32.96200000 46.57000000 15.18400000

H 33.14200000 48.69100000 16.62500000

H 33.29900000 50.94200000 15.43900000

H 33.17300000 48.92400000 11.63300000

H 33.42000000 46.78500000 12.83700000

C 33.29200000 51.52400000 12.85300000

H 34.33500000 51.92300000 12.66200000

H 32.71500000 51.48400000 11.96700000

H 32.82400000 52.33300000 13.40100000

C 33.61000000 35.34800000 12.14000000

C 33.70800000 34.18000000 12.87600000

C 33.29200000 33.01800000 12.38700000

C 32.61700000 32.97000000 11.13000000

C 32.55600000 34.13600000 10.37900000

C 33.00600000 35.30800000 10.84200000

H 33.85200000 36.30200000 12.72500000

H 34.23000000 34.22900000 13.80400000

H 33.53700000 32.06100000 12.88100000

H 31.97300000 34.07500000 9.46600000

H 32.74300000 36.28300000 10.39600000

C 32.03200000 31.76600000 10.42600000

H 31.63700000 30.97600000 11.15200000

H 31.22900000 32.11500000 9.74200000

H 32.80100000 31.24000000 9.85200000

C 28.88100000 33.79500000 14.31800000

C 29.35400000 35.06500000 13.96900000

C 29.19100000 36.19400000 14.78800000

C 28.43600000 36.10800000 15.96800000

C 27.89300000 34.81600000 16.22600000

C 27.99700000 33.71700000 15.46200000

H 29.03900000 32.97200000 13.62600000

H 30.01300000 35.06000000 13.05100000

H 29.56400000 37.18500000 14.36200000

H 27.35500000 34.68400000 17.15800000

H 27.56000000 32.83400000 15.86300000

C 28.26000000 37.30700000 16.90400000

H 29.02600000 37.34900000 17.69500000

H 28.26900000 38.18300000 16.25100000

H 27.20500000 37.33800000 17.21000000

C 34.13900000 41.02200000 8.39600000

C 34.57900000 39.76100000 8.07600000

C 35.12000000 39.48900000 6.82000000

C 35.02300000 40.40800000 5.78000000

C 34.62800000 41.66200000 6.05900000

C 34.17000000 41.98300000 7.35500000

H 33.58400000 41.13200000 9.36700000

H 34.77400000 39.07200000 8.91300000

H 35.42200000 38.44500000 6.67700000

H 34.36300000 42.41400000 5.38900000

H 33.70800000 42.97000000 7.62100000

C 35.32200000 40.09800000 4.32900000

H 35.75500000 40.87300000 3.72500000

H 35.70100000 39.05800000 4.18500000

H 34.33800000 39.99600000 3.81900000

C 24.95100000 45.26600000 11.47500000

C 24.67200000 43.94800000 11.22500000

C 23.88300000 43.61100000 10.16800000

C 23.39700000 44.64100000 9.28300000

C 23.84100000 45.95000000 9.41100000

C 24.56000000 46.24800000 10.55200000

H 25.48800000 45.42900000 12.49000000

H 24.96700000 43.15500000 11.90300000

H 23.47400000 42.53100000 10.02200000

H 23.46000000 46.78000000 8.86500000

H 24.79800000 47.30500000 10.63400000

C 22.51900000 44.29100000 8.12200000

H 22.97700000 43.69900000 7.37600000

H 21.57600000 43.76900000 8.51400000

H 22.24100000 45.20300000 7.57100000

C 36.22800000 46.28400000 7.90600000

C 35.02300000 46.84800000 8.40000000

C 34.28000000 46.14300000 9.35300000

C 34.66600000 44.83600000 9.73800000

C 35.86900000 44.31600000 9.26500000

C 36.67300000 45.07100000 8.45100000

H 36.90000000 46.83000000 7.24600000

H 34.73800000 47.87800000 8.21500000

H 33.34200000 46.52300000 9.75700000

H 36.38500000 43.43100000 9.67400000

H 37.64000000 44.57500000 8.11400000

C 33.74700000 44.15400000 10.70900000

H 33.75900000 44.54700000 11.69700000

H 32.69400000 44.15000000 10.48600000

H 34.00300000 43.05100000 10.68300000

C 27.18700000 49.03000000 1.64000000

C 27.86200000 47.86100000 1.93800000

C 27.17300000 46.61000000 1.83500000

C 25.83500000 46.54800000 1.44400000

C 25.16100000 47.75600000 1.20800000

C 25.84900000 48.96000000 1.33600000

H 27.73000000 49.94000000 1.59600000

H 28.89600000 47.79600000 2.16900000

H 27.78900000 45.72100000 2.06700000

H 24.10400000 47.63600000 0.87100000

H 25.32600000 49.86100000 1.18700000

C 25.08900000 45.24200000 1.40100000

H 24.31900000 45.42500000 0.63500000

H 25.77600000 44.46700000 1.04500000

H 24.65600000 44.95900000 2.33400000

C 25.49100000 41.80400000 0.06900000

C 24.91400000 41.16400000 -1.01900000

C 24.33200000 39.91100000 -0.79500000

C 24.29500000 39.31000000 0.50700000

C 24.79700000 40.03000000 1.59100000

C 25.30500000 41.32200000 1.34500000

H 26.11500000 42.68400000 0.06900000

H 25.09200000 41.53700000 -2.02700000

H 23.86900000 39.32800000 -1.60300000

H 24.88300000 39.71500000 2.62400000

H 25.71200000 41.92000000 2.16300000

C 23.75300000 37.88200000 0.73100000

H 22.71100000 37.80000000 0.89800000

H 23.99600000 37.22800000 -0.12200000

H 24.26500000 37.49200000 1.61100000

C 39.79000000 41.27700000 7.15800000

C 40.15500000 42.63200000 6.99400000

C 39.63100000 43.31100000 5.87500000

C 38.77500000 42.69800000 4.91500000

C 38.51900000 41.31300000 5.02900000

C 38.91300000 40.67900000 6.21700000

H 40.16300000 40.72700000 7.99400000

H 40.92100000 43.09900000 7.66000000

H 39.79400000 44.39000000 5.70700000

H 37.94800000 40.75900000 4.23300000

H 38.65500000 39.56200000 6.22800000

C 38.17800000 43.60300000 3.77900000

H 39.05000000 44.01700000 3.15000000

H 37.57000000 42.99700000 3.16400000

H 37.62000000 44.32300000 4.24800000

C 28.74200000 44.72800000 -1.45900000

C 27.68100000 45.64400000 -1.44100000

C 26.51300000 45.41200000 -2.15500000

C 26.39000000 44.22000000 -2.94400000

C 27.38000000 43.25600000 -2.83500000

C 28.53100000 43.45700000 -2.03400000

H 29.63600000 44.96300000 -0.86200000

H 27.74600000 46.60600000 -0.84500000

H 25.69000000 46.21800000 -2.08500000

H 27.17400000 42.32100000 -3.39300000

H 29.40100000 42.66500000 -2.05300000

C 25.18900000 44.02900000 -3.84200000

H 24.91200000 44.78900000 -4.53700000

H 25.21900000 43.09300000 -4.44400000

H 24.33000000 44.00800000 -3.16100000

C 27.93700000 30.33000000 10.79900000

C 28.11600000 31.72400000 10.75000000

C 27.24300000 32.54200000 11.43500000

C 26.20800000 32.02200000 12.23200000

C 26.11900000 30.63400000 12.37200000

C 26.96500000 29.83100000 11.57200000

H 28.64200000 29.73700000 10.25400000

H 28.96500000 32.12900000 10.25700000

H 27.45400000 33.62900000 11.46200000

H 25.43400000 30.21400000 13.09600000

H 26.89500000 28.67300000 11.61200000

C 25.49600000 33.03700000 13.14100000

H 24.46100000 32.88800000 12.92700000

H 25.57900000 32.81600000 14.19500000

H 25.61200000 34.16300000 12.95400000

C 40.39600000 43.29300000 10.83500000

C 40.04700000 42.48000000 11.92000000

C 38.87800000 42.72000000 12.61600000

C 38.14300000 43.84500000 12.31300000

C 38.53200000 44.69400000 11.26800000

C 39.57800000 44.36700000 10.46000000

H 41.38700000 43.01900000 10.39900000

H 40.69100000 41.54400000 12.19500000

H 38.60300000 42.08100000 13.51200000

H 38.04600000 45.65400000 11.10500000

H 39.71400000 45.08500000 9.60900000

C 36.97700000 44.32000000 13.15900000

H 36.31300000 43.60900000 13.69100000

H 37.33500000 44.89500000 14.03800000

H 36.39300000 44.99300000 12.51100000

C 33.65800000 31.56700000 4.17200000

C 32.99200000 31.45500000 5.41000000

C 33.29500000 30.46600000 6.27200000

C 34.45000000 29.61800000 5.99600000

C 35.07100000 29.67600000 4.76500000

C 34.72100000 30.67700000 3.80900000

H 33.50500000 32.43600000 3.50800000

H 32.20400000 32.17800000 5.62000000

H 32.87700000 30.25600000 7.24300000

H 35.86400000 28.98000000 4.59900000

H 35.33000000 30.71400000 2.88200000

C 34.87600000 28.57100000 6.98400000

H 34.73900000 28.98400000 7.98000000

H 34.23600000 27.67900000 6.99100000

H 35.97600000 28.31100000 6.94200000

C 30.68500000 46.65000000 5.90300000

C 29.51000000 46.03100000 5.58900000

C 28.21700000 46.56300000 5.89400000

C 28.14400000 47.88700000 6.42100000

C 29.32300000 48.52800000 6.68700000

C 30.59500000 48.01100000 6.40200000

H 31.66600000 46.21200000 5.67200000

H 29.62300000 45.00200000 5.25600000

H 27.36800000 45.92000000 5.86700000

H 29.40700000 49.59900000 6.96800000

H 31.52100000 48.59300000 6.58300000

C 26.75500000 48.49200000 6.55200000

H 26.32600000 48.62800000 5.52800000

H 26.08600000 47.78700000 7.13500000

H 26.73600000 49.49400000 6.99400000

C 30.48200000 39.77800000 7.22300000

C 30.53300000 40.10600000 8.60100000

C 30.46600000 41.36400000 9.08300000

C 30.48700000 42.40800000 8.10500000

C 30.35400000 42.07300000 6.71000000

C 30.33100000 40.76800000 6.28100000

H 30.42200000 38.71400000 6.94900000

H 30.67400000 39.28800000 9.32500000

H 30.31300000 42.86000000 5.89800000

H 30.34700000 40.52500000 5.20600000

C 30.60100000 41.61800000 10.55400000

H 30.28700000 40.78600000 11.28400000

H 31.70500000 41.63600000 10.77900000

H 30.16500000 42.56600000 10.79500000

O 30.59300000 43.71200000 8.47300000

H 29.79000000 44.11400000 8.19300000

o-cresol@toluene (within 8Å) 638ps

C 26.36800000 40.75800000 11.64900000

C 26.62500000 39.39400000 11.36100000

C 25.67800000 38.42000000 11.83100000

C 24.45700000 38.71100000 12.49900000

C 24.29300000 40.05800000 12.79800000

C 25.22900000 41.05700000 12.40200000

H 27.04600000 41.57000000 11.32300000

H 27.57100000 39.13900000 10.82100000

H 26.03400000 37.42100000 11.64200000

H 23.46700000 40.39400000 13.44400000

H 24.98900000 42.04300000 12.72200000

C 23.43200000 37.70700000 12.90300000

H 23.77400000 36.70100000 12.98300000

H 22.87100000 38.05000000 13.78200000

H 22.61600000 37.54400000 12.10900000

C 31.57900000 35.08800000 9.34700000

C 30.71300000 35.85300000 10.11000000

C 31.17800000 36.86700000 10.96300000

C 32.53300000 37.18200000 11.08400000

C 33.37100000 36.49300000 10.22900000

C 32.95100000 35.42100000 9.42000000

H 31.16400000 34.39300000 8.56900000

H 29.63400000 35.65800000 9.99200000

H 30.49900000 37.43100000 11.55800000

H 34.44800000 36.67200000 10.22900000

H 33.65400000 34.87500000 8.80600000

C 33.06900000 38.33100000 11.93500000

H 34.09400000 38.53400000 11.51100000

H 32.62700000 39.35900000 11.87000000

H 33.06700000 38.13800000 13.01300000

C 21.78100000 35.33700000 9.74800000

C 21.00900000 34.65400000 10.70000000

C 20.23800000 33.58100000 10.29600000

C 20.12400000 33.28300000 8.96200000

C 20.90900000 33.96700000 8.02800000

C 21.84800000 34.82400000 8.45800000

H 22.56100000 36.09900000 9.99900000

H 21.12300000 34.92600000 11.77600000

H 19.48600000 33.16600000 10.99900000

H 21.06100000 33.69900000 7.04200000

H 22.61600000 35.20500000 7.75600000

C 19.10000000 32.28600000 8.53900000

H 19.55100000 31.27800000 8.50500000

H 18.32800000 32.02800000 9.27600000

H 18.71000000 32.60800000 7.53000000

C 18.27200000 40.39100000 12.71100000

C 17.31500000 39.63700000 13.45900000

C 17.59100000 38.27500000 13.69600000

C 18.70500000 37.63300000 13.13400000

C 19.51800000 38.36600000 12.27800000

C 19.34200000 39.70300000 12.07000000

H 18.02100000 41.38300000 12.35200000

H 16.50300000 40.16600000 13.95500000

H 16.91100000 37.71700000 14.39300000

H 20.38000000 37.92900000 11.89300000

H 19.99400000 40.27600000 11.42300000

C 19.12400000 36.26200000 13.60300000

H 18.86700000 35.52200000 12.85000000

H 20.24200000 36.33600000 13.72500000

H 18.78900000 36.04100000 14.61700000

C 28.83800000 34.13400000 7.13700000

C 27.85900000 35.12800000 7.15600000

C 26.80100000 35.12900000 8.12300000

C 26.54100000 33.88300000 8.74300000

C 27.46000000 32.85000000 8.57800000

C 28.58400000 32.94400000 7.75500000

H 29.77500000 34.25000000 6.53700000

H 27.93900000 36.18600000 6.78300000

H 25.90100000 35.75100000 8.20300000

H 27.39000000 31.93600000 9.23600000

H 29.24400000 32.15100000 7.57500000

C 25.36800000 33.64900000 9.60000000

H 24.56900000 33.22700000 8.96900000

H 25.61200000 32.97700000 10.42700000

H 24.93700000 34.53300000 10.03600000

C 35.22800000 31.14600000 10.06500000

C 33.87300000 31.20600000 10.25500000

C 33.00800000 31.71500000 9.23500000

C 33.62000000 32.19000000 8.08800000

C 35.02900000 32.06400000 7.92100000

C 35.83300000 31.58700000 8.89800000

H 35.88100000 30.89000000 10.99300000

H 33.46600000 30.71300000 11.20800000

H 32.02200000 31.98500000 9.58200000

H 35.47500000 32.20800000 6.94200000

H 36.93100000 31.56700000 8.61000000

C 32.83000000 32.68900000 6.95300000

H 31.91400000 32.06900000 6.75700000

H 32.36400000 33.61000000 7.13300000

H 33.39800000 32.60700000 6.03500000

C 36.13900000 36.36300000 7.34000000

C 37.23500000 35.71400000 6.83300000

C 38.36100000 36.48200000 6.54400000

C 38.34000000 37.93100000 6.65100000

C 37.27700000 38.54000000 7.29700000

C 36.17100000 37.73400000 7.59800000

H 35.26100000 35.74500000 7.50000000

H 37.25300000 34.58300000 6.79500000

H 39.28400000 35.97200000 6.07100000

H 37.28500000 39.67100000 7.48400000

H 35.31500000 38.07400000 8.18500000

C 39.54000000 38.73700000 6.30200000

H 39.18000000 39.64900000 5.85800000

H 40.05100000 39.05100000 7.26800000

H 40.31800000 38.21600000 5.62500000

C 26.59800000 31.06400000 13.82000000

C 26.60000000 32.27200000 13.11000000

C 27.78000000 32.72500000 12.47200000

C 28.92000000 31.94300000 12.55900000

C 28.85900000 30.69200000 13.11700000

C 27.65100000 30.17700000 13.62100000

H 25.70500000 30.77200000 14.37100000

H 25.66500000 32.91700000 12.99400000

H 27.69900000 33.72800000 11.96400000

H 29.84400000 30.15500000 13.31300000

H 27.64600000 29.30300000 14.22300000

C 30.25400000 32.40000000 11.91900000

H 31.13400000 32.08000000 12.51800000

H 30.49900000 32.16300000 10.88000000

H 30.36600000 33.54400000 11.92400000

C 32.27400000 44.74200000 15.50100000

C 32.22700000 43.34300000 15.28600000

C 32.76800000 42.82800000 14.12600000

C 33.47400000 43.58200000 13.22300000

C 33.49400000 44.98000000 13.46200000

C 32.92200000 45.53700000 14.56100000

H 31.76200000 45.17000000 16.36600000

H 31.63800000 42.66900000 15.94200000

H 32.76300000 41.76600000 13.97200000

H 33.89000000 45.71900000 12.67700000

H 32.91300000 46.66800000 14.77900000

C 34.26000000 42.99200000 12.02900000

H 35.27700000 43.40500000 12.02000000

H 34.28500000 41.94700000 12.09300000

H 33.84000000 43.17000000 11.03400000

C 36.77600000 43.29400000 14.63600000

C 36.85900000 41.87900000 14.67100000

C 37.59800000 41.27400000 15.71800000

C 38.24400000 41.98000000 16.70700000

C 38.21300000 43.36200000 16.56400000

C 37.47800000 44.03500000 15.61100000

H 36.15300000 43.74300000 13.84600000

H 36.43500000 41.13600000 13.98000000

H 37.63300000 40.17600000 15.70800000

H 38.89800000 43.95000000 17.18000000

H 37.39400000 45.15200000 15.69200000

C 39.06100000 41.32200000 17.72600000

H 40.08400000 41.06600000 17.33500000

H 39.13800000 41.78100000 18.71900000

H 38.60100000 40.39000000 17.92900000

C 23.96100000 43.02200000 9.85800000

C 23.74200000 42.99600000 8.47200000

C 24.54200000 43.67400000 7.62100000

C 25.44100000 44.66500000 8.06600000

C 25.53700000 44.84500000 9.45200000

C 24.81300000 44.04100000 10.37700000

H 23.41700000 42.33500000 10.57100000

H 23.05300000 42.14300000 8.24200000

H 24.52600000 43.34000000 6.56800000

H 26.28400000 45.55500000 9.89800000

H 24.84300000 44.23500000 11.44500000

C 26.24300000 45.59300000 7.17700000

H 27.34700000 45.61700000 7.40800000

H 26.23900000 45.35700000 6.11700000

H 25.88500000 46.60900000 7.37000000

C 29.30600000 47.13000000 9.60500000

C 30.30000000 46.59800000 8.78700000

C 30.38700000 47.05300000 7.42200000

C 29.77300000 48.23800000 7.09900000

C 28.80200000 48.70100000 7.93000000

C 28.47100000 48.15000000 9.19800000

H 29.16200000 46.66600000 10.57500000

H 30.96000000 45.79000000 9.07200000

H 31.26900000 46.64700000 6.86300000

H 28.13800000 49.50300000 7.51600000

H 27.76000000 48.77400000 9.70600000

C 29.93600000 48.86000000 5.68300000

H 29.01000000 48.66600000 5.14300000

H 30.19700000 49.92200000 5.81000000

H 30.73600000 48.39500000 5.08700000

C 36.24300000 42.41700000 8.90700000

C 35.44600000 41.28600000 9.05300000

C 35.71600000 40.32500000 10.00400000

C 36.94400000 40.32700000 10.66900000

C 37.78100000 41.44200000 10.52400000

C 37.48400000 42.46700000 9.59500000

H 36.04400000 43.19900000 8.17600000

H 34.38700000 41.24100000 8.81000000

H 35.02200000 39.50200000 10.13500000

H 38.73400000 41.36800000 11.14300000

H 38.17600000 43.35400000 9.49400000

C 37.19100000 39.22800000 11.72400000

H 36.24400000 38.73300000 11.95300000

H 37.97600000 38.58900000 11.36100000

H 37.48000000 39.68000000 12.67200000

C 31.38300000 42.40800000 2.03600000

C 30.39000000 41.76300000 1.31000000

C 30.51500000 40.48400000 0.79500000

C 31.59200000 39.67800000 1.22300000

C 32.61600000 40.30700000 1.88000000

C 32.54100000 41.64800000 2.28400000

H 31.33600000 43.46300000 2.14400000

H 29.54500000 42.41400000 0.92300000

H 29.64600000 40.12900000 0.26300000

H 33.45100000 39.65200000 2.23800000

H 33.32800000 42.26300000 2.74600000

C 31.64500000 38.28000000 0.64200000

H 31.38800000 37.50900000 1.43900000

H 30.95900000 38.15000000 -0.13700000

H 32.66700000 38.14200000 0.21400000

C 26.70400000 40.13000000 18.09000000

C 26.27700000 39.47100000 19.25400000

C 25.34400000 40.06300000 20.10600000

C 24.88400000 41.33800000 19.78800000

C 25.26700000 41.96900000 18.57900000

C 26.15700000 41.31000000 17.72600000

H 27.42900000 39.56600000 17.48900000

H 26.65200000 38.43900000 19.48100000

H 24.83700000 39.59400000 20.94800000

H 24.75400000 42.90200000 18.27900000

H 26.30200000 41.80000000 16.78500000

C 23.85500000 41.97300000 20.66800000

H 23.44700000 41.41700000 21.50300000

H 24.07500000 42.96800000 21.11000000

H 23.03800000 42.08700000 19.98300000

C 27.84300000 46.73300000 2.38100000

C 27.88900000 47.84300000 1.62700000

C 27.88300000 47.81000000 0.20200000

C 27.48600000 46.57800000 -0.38200000

C 27.33000000 45.42100000 0.45100000

C 27.75400000 45.49500000 1.75500000

H 27.88600000 46.65600000 3.48300000

H 28.09700000 48.81800000 2.13100000

H 27.88900000 48.77000000 -0.37700000

H 27.08100000 44.46100000 0.02800000

H 27.52600000 44.61000000 2.32800000

C 27.25000000 46.37700000 -1.89400000

H 26.45200000 45.56900000 -2.01400000

H 27.06400000 47.38300000 -2.34300000

H 28.18600000 46.01500000 -2.34600000

C 41.83500000 44.87900000 11.37300000

C 40.45800000 44.72800000 11.43000000

C 39.93200000 43.58500000 12.06100000

C 40.77800000 42.72400000 12.76400000

C 42.16100000 42.86800000 12.72100000

C 42.69300000 43.95300000 12.00100000

H 42.34900000 45.74100000 10.83600000

H 39.70200000 45.36700000 10.93400000

H 38.86100000 43.51700000 12.23200000

H 42.97900000 42.20300000 13.00100000

H 43.78100000 44.01700000 12.03800000

C 40.15200000 41.47200000 13.35500000

H 40.63000000 40.59000000 12.97500000

H 40.22000000 41.55100000 14.43600000

H 39.10000000 41.45500000 13.18000000

C 24.73900000 35.11700000 4.78000000

C 24.97100000 35.81500000 3.57400000

C 23.88800000 36.03700000 2.66000000

C 22.56100000 35.76600000 3.02300000

C 22.37600000 35.03800000 4.19000000

C 23.41100000 34.70100000 5.04300000

H 25.50000000 34.84800000 5.55900000

H 25.99500000 36.06900000 3.34800000

H 24.09500000 36.70200000 1.74900000

H 21.36300000 34.88700000 4.58800000

H 23.30200000 33.93400000 5.83900000

C 21.40400000 36.17700000 2.18800000

H 20.44500000 35.87100000 2.73500000

H 21.39100000 37.29900000 2.06800000

H 21.25600000 35.67100000 1.20200000

C 33.27600000 43.91300000 5.96000000

C 32.53400000 44.65100000 5.02700000

C 31.18000000 44.47600000 4.95900000

C 30.47500000 43.57900000 5.77400000

C 31.26800000 42.98900000 6.76100000

C 32.66000000 43.05800000 6.83700000

H 34.31000000 44.00100000 5.87000000

H 33.11900000 45.23500000 4.29600000

H 30.70400000 45.00400000 4.13200000

H 30.77300000 42.19700000 7.31300000

H 33.17900000 42.62500000 7.70200000

C 29.05900000 43.30900000 5.51600000

H 28.57700000 44.06600000 4.89900000

H 28.89500000 42.34200000 5.10700000

H 28.51700000 43.31500000 6.46800000

C 30.53900000 42.39500000 9.80100000

C 29.24000000 42.29900000 9.27000000

C 28.83400000 41.02200000 8.74900000

C 29.70700000 39.89200000 8.80400000

C 30.88800000 39.96600000 9.44700000

C 31.29400000 41.23800000 9.91500000

H 30.94400000 43.31900000 10.24600000

H 28.47100000 43.07700000 9.21800000

H 31.65500000 39.24500000 9.38600000

H 32.25200000 41.34500000 10.34200000

C 27.51500000 41.00900000 8.00900000

H 26.72700000 40.52200000 8.55200000

H 27.19100000 42.04300000 7.89500000

H 27.67100000 40.50100000 7.03300000

O 29.23900000 38.76600000 8.21800000

H 29.93900000 38.12300000 8.21500000

o-cresol@toluene (within 4Å) 250ps

C 16.40600000 39.52300000 5.71500000

C 17.72200000 39.23900000 6.28700000

C 18.43300000 38.10600000 5.86400000

C 17.97900000 37.36300000 4.77800000

C 16.72400000 37.62500000 4.24800000

C 15.93900000 38.68000000 4.66000000

H 15.95600000 40.51800000 5.77200000

H 18.33300000 39.84700000 6.99500000

H 19.44800000 37.84700000 6.29800000

H 16.37700000 36.95400000 3.38500000

H 14.93100000 38.88600000 4.17100000

C 18.86100000 36.45200000 4.04500000

H 18.35500000 36.13800000 3.14200000

H 19.83800000 36.96500000 3.79300000

H 18.99400000 35.58100000 4.75300000

C 25.47100000 46.43400000 9.53800000

C 25.80300000 46.58900000 8.19000000

C 24.83800000 46.26700000 7.20000000

C 23.62500000 45.71600000 7.49100000

C 23.32000000 45.46400000 8.77200000

C 24.26100000 45.77500000 9.75900000

H 26.31100000 46.55300000 10.25500000

H 26.75100000 47.07000000 7.90200000

H 25.12700000 46.42900000 6.13900000

H 22.41900000 44.92400000 8.99400000

H 23.95700000 45.44900000 10.74100000

C 22.71500000 45.24500000 6.33400000

H 22.79900000 45.90300000 5.51400000

H 23.05000000 44.34200000 5.86500000

H 21.68200000 45.22400000 6.66800000

C 20.30000000 38.30600000 -1.79000000

C 21.06900000 39.45400000 -2.04600000

C 20.28100000 40.57600000 -2.48700000

C 18.90400000 40.65600000 -2.25200000

C 18.22500000 39.47800000 -1.98600000

C 18.94100000 38.33200000 -1.74400000

H 20.80700000 37.38100000 -1.40800000

H 22.21000000 39.45100000 -2.01000000

H 20.87700000 41.44900000 -2.69000000

H 17.12700000 39.45800000 -1.92000000

H 18.44100000 37.30600000 -1.53800000

C 18.10000000 41.97800000 -2.41900000

H 18.67900000 42.83800000 -2.86400000

H 17.32200000 41.88700000 -3.14900000

H 17.69200000 42.25100000 -1.48700000

C 25.26000000 39.38700000 7.38200000

C 25.16100000 40.79300000 7.47000000

C 23.97300000 41.46600000 7.25800000

C 22.82100000 40.75000000 6.86200000

C 22.93600000 39.32400000 6.68100000

C 24.11200000 38.68000000 7.10000000

H 26.25200000 39.05700000 7.72700000

H 26.10400000 41.36800000 7.67200000

H 24.05800000 42.52000000 7.33100000

H 22.03000000 38.74600000 6.42000000

H 24.19600000 37.59300000 7.04200000

C 21.48300000 41.36700000 6.43900000

H 21.56700000 41.71400000 5.41600000

H 20.60700000 40.73600000 6.62500000

H 21.33600000 42.36300000 6.99100000

C 19.89600000 33.72500000 8.40200000

C 20.64700000 32.60100000 8.15300000

C 21.77000000 32.72100000 7.32900000

C 22.04400000 33.85900000 6.55100000

C 21.14400000 34.87100000 6.67000000

C 20.13900000 34.86700000 7.65400000

H 19.02500000 33.79300000 9.09400000

H 20.45700000 31.69000000 8.74600000

H 22.33900000 31.76000000 7.24700000

H 21.37600000 35.80100000 6.03600000

H 19.54600000 35.79600000 7.82200000

C 23.17900000 33.79800000 5.58900000

H 22.87600000 33.44300000 4.58900000

H 23.69000000 34.75600000 5.50100000

H 23.88700000 33.04500000 5.86500000

C 19.74200000 44.47900000 0.64900000

C 18.44000000 45.06000000 0.89200000

C 17.77400000 45.84200000 -0.01300000

C 18.26100000 45.98800000 -1.29100000

C 19.52200000 45.36300000 -1.55900000

C 20.31800000 44.74900000 -0.60100000

H 20.14700000 43.84200000 1.47000000

H 18.05500000 44.86900000 1.92700000

H 16.71600000 46.03900000 0.17400000

H 20.03800000 45.64300000 -2.52100000

H 21.24100000 44.17200000 -0.97200000

C 17.64300000 46.72100000 -2.44900000

H 17.19100000 46.04600000 -3.15700000

H 16.98500000 47.50100000 -2.08800000

H 18.36000000 47.18300000 -3.13300000

C 22.14400000 41.83700000 0.81000000

C 22.52100000 40.52000000 1.19900000

C 23.19400000 40.32600000 2.44600000

C 23.69700000 41.42300000 3.14200000

C 23.39200000 42.72600000 2.71400000

C 22.66800000 42.86400000 1.58300000

H 21.73900000 41.97100000 -0.19900000

H 22.16900000 39.66300000 0.58200000

H 23.74300000 43.59200000 3.30400000

H 22.49400000 43.81400000 1.17900000

C 23.31800000 38.90800000 2.89600000

H 24.27800000 38.89300000 3.41800000

H 22.63100000 38.63200000 3.75600000

H 23.24700000 38.16300000 2.14000000

O 24.43400000 41.41100000 4.31000000

H 24.50700000 40.49100000 4.59200000

o-cresol@toluene (within 4Å) 750ps

C 36.09200000 40.24600000 9.12300000

C 36.39900000 41.07800000 10.24500000

C 37.01600000 40.41800000 11.31300000

C 37.37900000 39.09700000 11.31400000

C 37.09600000 38.39200000 10.19900000

C 36.42700000 38.89200000 9.11600000

H 35.70800000 40.71100000 8.17700000

H 35.95600000 42.05700000 10.36200000

H 37.27000000 40.98100000 12.24300000

H 37.33600000 37.31400000 10.31100000

H 36.25700000 38.36300000 8.16900000

C 38.15300000 38.46000000 12.47800000

H 37.51400000 37.73700000 12.98800000

H 38.95000000 37.94300000 11.98200000

H 38.66700000 39.12000000 13.22400000

C 39.91300000 43.35400000 -1.29900000

C 39.22200000 43.88800000 -2.35300000

C 37.94600000 44.50000000 -2.18000000

C 37.44000000 44.67200000 -0.84300000

C 38.20400000 44.17900000 0.21600000

C 39.40900000 43.53600000 -0.00300000

H 40.75600000 42.73200000 -1.44800000

H 39.74400000 43.77200000 -3.31200000

H 37.28400000 44.82000000 -3.04500000

H 37.75000000 44.12800000 1.24000000

H 40.05700000 43.13200000 0.76200000

C 36.00000000 44.96300000 -0.61600000

H 35.68000000 45.63900000 -1.41200000

H 35.39300000 44.00400000 -0.79600000

H 35.88500000 45.30200000 0.43500000

C 32.76000000 39.76400000 9.03100000

C 32.21900000 40.32300000 7.94200000

C 31.60600000 41.59300000 7.99000000

C 31.76600000 42.32800000 9.17300000

C 32.33600000 41.73900000 10.23500000

C 32.88600000 40.49800000 10.19300000

H 33.01500000 38.72600000 9.02800000

H 32.34300000 39.78500000 6.95200000

H 31.01000000 42.06100000 7.20700000

H 32.55400000 42.36100000 11.12700000

H 33.36700000 39.85300000 11.10000000

C 31.14300000 43.76600000 9.27000000

H 31.54500000 44.30700000 8.46300000

H 30.05000000 43.83800000 9.26700000

H 31.34600000 44.13500000 10.28400000

C 29.50500000 42.27200000 -1.17500000

C 28.94200000 41.77600000 0.02100000

C 29.81100000 41.23700000 0.97100000

C 31.11500000 40.99700000 0.82100000

C 31.72600000 41.66700000 -0.26700000

C 30.88200000 42.18600000 -1.26700000

H 28.82200000 42.74500000 -1.90600000

H 27.86500000 41.97900000 0.24900000

H 29.27800000 40.75800000 1.87900000

H 32.81400000 41.69800000 -0.47400000

H 31.22100000 42.76200000 -2.13300000

C 31.94500000 40.30100000 1.88700000

H 32.46100000 39.47000000 1.44400000

H 32.79300000 40.96600000 2.24400000

H 31.43600000 39.99900000 2.84800000

C 33.90900000 37.94300000 -0.51900000

C 34.64700000 38.36000000 0.60500000

C 35.42100000 37.34200000 1.23200000

C 35.59200000 36.08700000 0.70500000

C 34.94200000 35.76700000 -0.48200000

C 33.97000000 36.62700000 -1.04300000

H 33.18200000 38.63300000 -0.92500000

H 34.58800000 39.44600000 0.93300000

H 35.69600000 37.69800000 2.25300000

H 35.04200000 34.83400000 -0.92000000

H 33.48100000 36.34300000 -1.96000000

C 36.45100000 35.05100000 1.46200000

H 37.24600000 35.50700000 2.12600000

H 35.93800000 34.22700000 2.07400000

H 37.08100000 34.47000000 0.63400000

C 42.25200000 39.98400000 8.33500000

C 43.04500000 39.40900000 7.24600000

C 42.57300000 38.34200000 6.47700000

C 41.31700000 37.80100000 6.70300000

C 40.59000000 38.26100000 7.84400000

C 41.07800000 39.29500000 8.61400000

H 42.70100000 40.98700000 8.70500000

H 43.97200000 39.88600000 6.89700000

H 43.19300000 38.04200000 5.55300000

H 39.55000000 37.82600000 8.20500000

H 40.41000000 39.60900000 9.48700000

C 40.64600000 36.80900000 5.77300000

H 40.92400000 35.75100000 6.07000000

H 39.56200000 36.80900000 5.83700000

H 41.02600000 37.04700000 4.77000000

C 35.85800000 43.45800000 6.44300000

C 35.55600000 44.60900000 5.68800000

C 36.55000000 45.24400000 4.91400000

C 37.86600000 44.78000000 4.96300000

C 38.16400000 43.69700000 5.80100000

C 37.21200000 43.10700000 6.58500000

H 35.03300000 43.05400000 7.07600000

H 34.50200000 44.92400000 5.51400000

H 36.37200000 46.18800000 4.34300000

H 39.19200000 43.32000000 5.76300000

H 37.51500000 42.18500000 6.98600000

C 38.87300000 45.47300000 4.11200000

H 39.73900000 44.85100000 3.87900000

H 38.55900000 45.90000000 3.09000000

H 39.43500000 46.26000000 4.68300000

C 34.04000000 34.64800000 8.36900000

C 34.92200000 33.59100000 7.96100000

C 35.89400000 33.93500000 6.98800000

C 35.85200000 35.16500000 6.31700000

C 34.93900000 36.14300000 6.67900000

C 34.04500000 35.86500000 7.67100000

H 33.38100000 34.46500000 9.19600000

H 34.95500000 32.64400000 8.58100000

H 36.50900000 33.10400000 6.51800000

H 34.95200000 37.12200000 6.28200000

H 33.27800000 36.62100000 7.86900000

C 36.96800000 35.48200000 5.33800000

H 37.86100000 35.07000000 5.87900000

H 36.79100000 34.94800000 4.35400000

H 37.05300000 36.54900000 5.08000000

C 38.71200000 40.46000000 4.32500000

C 37.92900000 41.31300000 3.52700000

C 36.59500000 41.52200000 3.76500000

C 35.93400000 40.65700000 4.60000000

C 36.68000000 39.72400000 5.36900000

C 38.05400000 39.69100000 5.30500000

H 39.78400000 40.44400000 4.35300000

H 38.48900000 41.94000000 2.82500000

H 36.23400000 39.03000000 6.08600000

H 38.56300000 39.10500000 6.09600000

C 35.82900000 42.51300000 2.84000000

H 35.83000000 42.18400000 1.79000000

H 34.74000000 42.70500000 3.19800000

H 36.29100000 43.52200000 2.89700000

O 34.56800000 40.65200000 4.76600000

H 34.24700000 41.55900000 4.49700000

o-cresol@water 850ps

O 10.81500000 11.22800000 14.87000000

H 10.76200000 11.62800000 15.78300000

H 11.40900000 10.49600000 14.97600000

O 7.03000000 12.54600000 13.11400000

H 7.52400000 12.05000000 13.76600000

H 7.61400000 12.69100000 12.34000000

O 7.55000000 1.95100000 19.91300000

H 7.97100000 1.84900000 19.04600000

H 6.66200000 2.17300000 19.62400000

O 5.10300000 5.59000000 12.74400000

H 5.90600000 6.11500000 12.72100000

H 5.04100000 5.25500000 11.83900000

C 5.96100000 7.81600000 15.19400000

C 4.71200000 7.75700000 15.79700000

C 4.56300000 7.09600000 17.02600000

C 5.64900000 6.43000000 17.62300000

C 6.89100000 6.38100000 16.94300000

C 7.02200000 7.09300000 15.78900000

H 6.09800000 8.57500000 14.41100000

H 3.95500000 8.40700000 15.41600000

H 3.57900000 7.22600000 17.58200000

H 8.08500000 7.19500000 15.42500000

C 8.04900000 5.62400000 17.56300000

H 8.53800000 6.14700000 18.39600000

H 7.83900000 4.58800000 17.85100000

H 8.79200000 5.47500000 16.75200000

O 5.35500000 5.85100000 18.84300000

H 5.98100000 5.08700000 18.94600000

o-cresol@water 450ps

O 6.28700000 9.40000000 4.65200000

H 5.71300000 9.79000000 5.36000000

H 6.46400000 8.53500000 5.06900000

O 14.26500000 8.97700000 13.55600000

H 13.83100000 8.10600000 13.70500000

H 14.20900000 9.03700000 12.57000000

O 17.47400000 9.60000000 8.54800000

H 16.79300000 9.11400000 8.01500000

H 17.20600000 10.55800000 8.28100000

O 10.29900000 10.83100000 16.37400000

H 10.78500000 11.74700000 16.35900000

H 10.07500000 10.86800000 15.40400000

O 10.52800000 15.33400000 11.86100000

H 10.07800000 14.99100000 11.07600000

H 11.10200000 16.02100000 11.55200000

O 7.55900000 15.43800000 6.65700000

H 6.78800000 14.80100000 6.60500000

H 8.07100000 14.98200000 7.35700000

O 2.73200000 12.31300000 4.62500000

H 1.84500000 11.94900000 4.42400000

H 3.18600000 12.14200000 3.78200000

O 4.82700000 9.70800000 14.83300000

H 5.08100000 8.73200000 14.83400000

H 5.81300000 10.05100000 14.82000000

O 13.65000000 11.68900000 10.31500000

H 14.42100000 11.32200000 10.79100000

H 13.95300000 12.13700000 9.48200000

O 5.23300000 5.33800000 11.78100000

H 6.03800000 5.53500000 11.27300000

H 4.80600000 6.20300000 11.94800000

O 7.42400000 17.92200000 5.28300000

H 7.61100000 16.97300000 5.57000000

H 6.51300000 17.68100000 4.96100000

O 13.32300000 7.12600000 8.32800000

H 12.82800000 7.88900000 7.84800000

H 13.44900000 7.61300000 9.19600000

O 3.49500000 15.00900000 13.40300000

H 4.06900000 15.73200000 13.07000000

H 3.48000000 15.24000000 14.35400000

C 8.41300000 9.37700000 8.88600000

C 9.74500000 9.10300000 9.28500000

C 10.51800000 10.12000000 9.79300000

C 10.03400000 11.43600000 9.83700000

C 8.71000000 11.68700000 9.44300000

C 7.87700000 10.65600000 9.02600000

H 7.78000000 8.71100000 8.41700000

H 10.06500000 8.05900000 9.05300000

H 11.61700000 9.99800000 10.07300000

H 6.97800000 10.87500000 8.42700000

C 8.03600000 13.05600000 9.53800000

H 7.18200000 13.05800000 8.82500000

H 7.50200000 13.06200000 10.54600000

H 8.66500000 13.87400000 9.44600000

O 10.87200000 12.41500000 10.23500000

H 11.72000000 12.01000000 10.44200000
